# Hepatoblastoma: Derived Exosomal LncRNA NEAT1 Induces BMSCs Differentiation into Tumor-Supporting Myofibroblasts via Modulating the miR-132/MMP9 Axis

**DOI:** 10.1155/2022/7630698

**Published:** 2022-03-08

**Authors:** Yu Hu, Hongyan Zai, Wei Jiang, Zhenglin Ou, Yuanbing Yao, Qin Zhu

**Affiliations:** Department of General Surgery, Xiangya Hospital, Central South University, Changsha, Hunan 410008, China

## Abstract

**Background:**

Hepatoblastoma (HB) is the most common malignant tumor of the liver. MMP9 plays an essential role in HB. The purpose of our study was to screen for differentially expressed lncRNAs and miRNAs that targeted MMP9. Based on this, the role of lncRNA NEAT1/miR-132/MMP9 in HB and the mechanisms involved were discussed.

**Methods:**

Bioinformatics analysis was used to screen the differentially expressed lncRNAs and miRNAs targeting MMP9. Exosomes were extracted from HB cells and normal liver cells for characterization and identification. Exosome uptake assay was conducted to determine whether exosomes were absorbed by bone marrow stromal cells (BMSCs). *α*-SMA, fibronectin, and s-100 expressions in tissues and cells were detected by IHC and ICC. lncRNA XIST, lncRNA NEAT1, miR-132, and MMP9 expressions were characterized by qRT-PCR. Western blot was performed to measure MMP9, *α*-SMA, and s-100 expressions. Flow cytometry was used to stain *α*-SMA, s-100. Bioinformatics and dual-luciferase reporter assay were applied to verify the interaction between lncRNA NEAT1 and miR-132, and miR-132 and MMP9. The effect of lncRNA NEAT1 on the development of HB in nude mice was studied.

**Results:**

Differentially expressed lncRNA NEAT1/miR-132/MMP9 was obtained through bioinformatics analysis and cell verification. HB-derived exosomal lncRNA NEAT1 regulated miR-132 and MMP9 expression in BMSCs. In addition, HB-derived exosomal lncRNA NEAT1 promoted BMSCs differentiation toward invasive myofibroblast via miR-132/MMP9 axis. LncRNA NEAT1 regulated MMP9 through miR-132. Tumor formation experiments in nude mice showed that HB-derived exosomal lncRNA NEAT1 could affect the development of HB.

**Conclusion:**

HB-derived exosomal lncRNA NEAT1 induced BMSCs differentiation into tumor-supporting myofibroblasts via modulating miR-132/MMP9 axis, which provided a new target for HB treatment.

## 1. Introduction

Hepatoblastoma (HB) is a rare malignant tumor derived from pluripotent stem cells, most common in children and mainly occurs in the first two years of life [1, 2]. HB is associated with different histological subtypes and different degrees of clinical aggressiveness and presentation [3]. Currently, the main treatment methods include chemotherapy, surgical resection and transplantation [4]. Chemotherapy drugs, including cisplatin, have been applied in the treatment strategy of HB [5]. The study has shown that the outcome of patients with HB depends on the resectability of the tumor and the presence of metastatic disease [6]. Currently, surgery is the main treatment for HB, and in developed countries, the 5-year overall survival rate is about 80% [7, 8]. Due to the genetic heterogeneity of HB, its therapeutic target has not been defined. Therefore, there is an urgent need to explore the pathogenesis of HB and develop new therapeutic targets to improve the clinical outcome of HB patients.

Cell-to-cell communication between primary tumor cells via extracellular vesicles (exosomes and microvesicles) and the microenvironment of distal organs is essential for the formation and metastasis of premetastatic niches (PMNs) [9]. The exosome is a functional carrier that mediates information exchange between cells and plays a role between cells by delivering functional active substances (such as proteins, lipids, RNA molecules, and circulating DNA) [10, 11]. Exosomes play an essential role in the occurrence and evolution of HB, including promoting the formation of the liver cancer microenvironment, enhancing tumor invasion and metastasis, enhancing angiogenesis, and participating in tumor immunosuppression and tumor chemoradiotherapy resistance [12, 13]. The liver itself has a strong regenerative ability due to the existence of stem cells [14]. The study has shown that exosomes of cancer cells can promote the differentiation of stem cells to cancer stem cells and myofibroblasts with the invasive ability [15]. In this study, relevant miRNAs in cancer cell exosomes were screened, and their effects on stem cell differentiation were investigated. By changing the expression of lncRNA in the exosomes of cancer cells, the canceration of stem cells can be inhibited.

Mesenchymal stem cells (MSCs) are significant components in the tumor microenvironment that promote tumor progression [16]. Tumor cells secrete exosomes containing bioactive molecules, such as lncRNAs, miRNAs, and proteins, in the pretumorigenic form of reprogrammed primary MSCs [17]. The study has reported that the exosome lncRNA X26nt secreted by gastric cancer increases angiogenesis and vascular permeability by targeting VE-cadherin [18]. Aggressive myofibroblasts are characterized by *α*-SMA expression and play an essential role in tumor progression and tissue fibrosis [19]. The study has shown that some cancer-derived exosomes can trigger an increase in *α*-SMA protein expression, and others can differentiate fibroblasts into myofibroblasts [20]. Matrix metalloproteinase (MMP) 9 has been reported to be elevated in HB [21]. Based on this, we evaluated the effect of HB-derived exosomal lncRNAs on promoting differentiation of bone marrow stromal cells (BMSCs) into myofibroblasts and explored its functional axis through the miRNA/MMP9 signaling pathway.

Therefore, in this study, we screened miRNAs and lncRNAs with significant differences in HB through bioinformatics analysis. The results showed that among the miRNAs in HB, the expression difference of hsa-miR-132 was the largest. Based on this, the combined lncRNA NEAT1 was screened. Therefore, we focused on the regulatory mechanism of lncRNA NEAT1/miR-132/MMP9 in HB. We found that HB-derived exosomal lncRNA NEAT1 induced BMSCs to differentiate into tumor-supporting myofibroblasts by regulating the miR-132/MMP9 axis, which might benefit an innovative therapeutic strategy for the treatment of HB patients.

## 2. Materials and Methods

### 2.1. Clinical Samples

HB tissues and paracancerous tissues samples (*n* = 15) were collected from patients diagnosed as HB by iconography, serological, or histopathological examination in Xiangya Hospital between September 2020 and March 2021. We have obtained the subjects' written informed consent prior to the study and obtained the approval from the Human Research Ethics Committee of Xiangya Hospital (AF/SQ202104800).

### 2.2. Bioinformatics Analysis

Clinical samples were divided into HB tissues and paracancerous tissues. Data were obtained from GEO database cohorts GSE132219 and GSE20971, and the data types were RNA microarray and miRNA microarray. *R* package was used to download GEOquery GSE132219 and GSE20971 chip original expression data. *R* package DEseq2 was performed to screen differentially expressed miRNAs, lncRNAs, and mRNAs, and selection criteria were |logFC| > 1 and *P* value < 0.05. Then we mapped the volcano. Target genes with differential expression of miRNAs were predicted using miTARbase database, and the Venn diagram was drawn to screen miRNAs specifically binding to MMP9. EncoRI database was used to predict the target lncRNAs differentially expressed miRNAs, and the screening criterion was the number of clip experiments >7. Box diagrams of differentially expressed miRNAs were drawn. Co-expressed DE lncRNAs and DE mRNAs were screened according to the correlation coefficient, miRNA-mRNA-lncRNA network was plotted using Cytoscape, and the lncRNA-miRNA-MMP9 regulatory subnetwork was extracted.

### 2.3. Cell Culture and Treatment

Human normal liver cells WRL68 (bio-53604) and HB cells HepG2 (bio-105877), HB611 (bio-73286), and Huh-6 (bio-73060) were purchased from Biobw and cultured in DMEM medium (D5796, Sigma) containing 10% FBS at 37°C and 5% CO_2_. Exosomes were taken to characterize their morphology. BMSCs were purchased from Yaji Biological and cultured in a cell-free DMEM medium at 37°C and 5% CO_2_. Exosome stimulation of BMSCs was divided into Control, BMSCs + liver cell exosome, and BMSCs + HB cell exosome groups. Cells were collected 7 days later for follow-up tests. To investigate the role of lncRNA NEAT1 in HB, knockdown and overexpression of lncRNA NEAT1 were performed. At the same time, MMP9 was knocked down to investigate whether lncRNA NEAT1 influenced BMSCs' differentiation to invasive myofibroblasts via the miR-132/MMP9 axis. They were grouped as Control, BMSC + siNC exos, BMSC + siNEAT1 exos, BMSC + oeNC exos, BMSC + oeNEAT1 exos, BMSC + oeNEAT1 exos + siNC, BMSC + oeNEAT1 exos + siMMP9 groups. BMSCs were co-cultured with exosomes. siNEAT1 and the corresponding negative control siNC were synthesized by Sangon Biotech (Shanghai, China). According to the instructions, the siNC and siNEAT1 sequences were transfected into the cells using Lipofectamine 3000 (Invitrogen) reagent. Then we extracted exosomes from cells. To overexpress lncRNA NEAT1, lncRNA NEAT1 sequences were ligated with the LV003 vector. According to the instructions, the oeNC and oeNEAT1 sequences were transfected into the cells using Lipofectamine 3000 reagent. Then we extracted exosomes from cells. siMMP9 was synthesized by Sangon Biotech, and the corresponding negative control siNC was used to knock down MMP9 expression. According to the instructions, the siNC and siMMP9 sequences were transfected into the cells using Lipofectamine 3000 reagent.

### 2.4. Exosome Extraction and Identification

Exosomes were extracted from the cell culture supernatant using an exosome extraction kit (EXOQ5A-1, SBI) according to the instructions. BMSCs were cultured in a cell-free DMEM medium, and the supernatant was collected from 48 to 72 h to extract exosomes for subsequent detection. Extracted exosomes were resuspended in PBS. The morphology and size of exosomes were characterized by transmission electron microscopy (TEM) and nanoparticle tracking analysis (NTA). CD9 (ab927266, 1 : 2000, Abcam), CD63 (ab217345, 1 : 1000, Abcam), CD81 (ab109201, 1 : 2000, Abcam), and CD326 (ab213500, 1 : 1000, Abcam) were used in Western blot analysis. *β*-Actin (66009-1-Ig, 1 : 5000, Proteintech) was an internal reference gene.

### 2.5. Exosome Uptake Assay

The sterile slides were placed in the cell culture plates, and the pDCs obtained by magnetic bead separation were seeded into the 12-well plates with the slides placed in advance according to 2 × 10^4^/mL. The culture was maintained overnight in 1640 medium with 2% FBS. A small volume of 20 *μ*g exosome solution was resuspended with 250 *μ*L Diluent C solution. The PKH67 dye solution was prepared, and the exosome solution was mixed with the PKH67 dye solution. After incubation for 4 min, 420 *μ*L 10% BSA was added to bind the excess PKH67 dye. A 1:5 volume of Exoquick-TC solution was added and mixed upside down and left standing at 4°C overnight. Then they were centrifuged at 1500*g* for 30 min. The supernatant was discarded, and the precipitation was resuspended with 50 *μ*L PBS. PKH67 labeled exosomes were added and co-incubated with plasma dendritic cells for 6 h. The slides were taken out, washed twice with PBS, fixed with 4% paraformaldehyde for 30 min, and washed three times with PBS. The phalloidine was diluted with PBS into the working solution (1 : 100 dilution), incubated in the dark for 30 min, and washed with PBS 3 times. A small volume of 1 mL DAPI (1 *μ*g/mL) was added and incubated for 10 min in the dark at 37°C. Then they were washed with PBS three times and observed under a fluorescence microscope.

### 2.6. Immunohistochemistry

The expression of *α*-SMA, fibronectin, and s-100 in HB and paracancerous tissues were detected by immunohistochemistry (IHC). The slices were roasted at 60°C for 12 h, and the slices were dewaxed to water. The antigens were repaired by heat. About 1% periodate acid was added, and the endogenous enzymes were inactivated at room temperature for 10 min. Appropriately diluted primary antibody *α*-SMA (14395-1-AP, 1 : 100, Proteintech), fibronectin (66042-1-Ig, 1 : 100, Proteintech), and s-100 (16105-1-AP, 1 : 100, Proteintech) were incubated at 4°C overnight. Then they were washed with PBS 5 min for three times. The secondary antibody was incubated at 37°C for 30 min and washed with PBS 5 min three times. DAB was used for color development. Sections were re-stained with hematoxylin for 5 min. All levels of alcohol were dehydrated for 5 min. They were treated with xylene and then sealed with neutral gum and observed under a microscope. A 400-fold field of view was taken, and the IOD analysis was performed by Image Pro Plus 6.0 image analysis software. The relative expression levels of *α*-SMA, fibronectin and s-100 were represented by the average optical density (IOD of the positive area under the field of view/tissue area under the field of view).

### 2.7. Immunocytochemistry

The expression of *α*-SMA, fibronectin, and s-100 in BMSCs was detected by immunocytochemistry (ICC). The slide was fixed with 4% paraformaldehyde for 30 min and washed with PBS 5 min for three times. About 3% H_2_O_2_ was added at room temperature for 10 min to inactivate endogenous enzymes. They were washed with PBS 5 min for three times. Appropriately diluted primary antibodies *α*-SMA (14395-1-AP, 1 : 50, Proteintech), fibronectin (66042-1-Ig, 1 : 50, Proteintech), and s-100 (16105-1-AP, 1 : 50, Proteintech) were incubated at 4°C overnight. Then they were washed with PBS 5 min for three times. The secondary antibody was incubated at 37°C for 30 min and washed with PBS 5 min for 3 times. DAB was used for color development, hematoxylin was re-dyed for 5 to 10 min, washed with distilled water, and PBS returned to blue. All levels of alcohol were dehydrated for 5 min. After taking it out, it was placed in xylene for 10 min. Then it was sealed with neutral gum and observed under microscope.

### 2.8. Quantitative Real-Time PCR

The expression of lncRNA NEAT1, lncRNA XIST, miR-132, and MMP9 was characterized by Quantitative Real-Time polymerase chain reaction (qRT-PCR). Total RNA was extracted by Trizol method, and the cDNA reverse transcription kit (4368814, Invitrogen) was used to reverse transcript RNA into cDNAs. The relative gene expression was tested on the ABI 7900 system using SYBR Green qPCR Mix (Invitrogen). *β*-Actin and U6 were used as the reference gene, and the relative gene level was calculated using 2^−ΔΔCT^ method. Primer sequences used in this study were as follows: lncRNA NEAT1-F: CCTGCCTTCTTGTGCGTTT, lncRNA NEAT1-R: TAGCACAACACAATGACACCCT; lncRNA XIST-F: ACTGCCACCCATATATAAGCTA, lncRNA XIST-R: AGTAATCACCATTCAGTAAGCCA; miR-132-F: TAACAGTCTACAGCCATGGTCG, miR-143-R: ACCGTGGCTTTCGATTGTTACT; MMP9-F: CTGAAGGCCATGCGAACCCCA, MMP9-R: GCAAAGGCGTCGTCAATCACC; U6-F: CTCGCTTCGGCAGCACA, U6-R: AACGCTTCACGAATTTGCGT; *β*-actin-F: ACCCTGAAGTACCCCATCGAG, *β*-actin-R: AGCACAGCCTGGATAGCAAC.

### 2.9. Western Blot

Total proteins were extracted from tissues and cells using RIPA lysis buffer according to the instructions, and the protein was quantitated according to the BCA protein assay kit. The loading buffer of SDS-PAGE was mixed, and the mixture was heated in a boiling water bath at 100°C for 5 min. The proteins were adsorbed on the PVDF membrane through gel electrophoresis. The membranes were sealed with 5% skim milk solution at room temperature for 2 h, and then incubated with primary antibodies MMP9 (AB38898, 1 : 1000, Abcam), *α*-SMA (14395-1-AP, 1 : 2000, Proteintech), fibronectin (66042-1-IG, 1 : 100, Proteintech), s-100 (16105-1-AP, 1 : 1000, Proteintech), and *β*-actin (66009-1-Ig, 1 : 5000, Proteintech) at 4°C overnight. After incubation, the membranes were washed with TBST at room temperature. The secondary antibody HRP goat antimouse IgG (SA00001-1, 1 : 5000, Proteintech) or HRP goat antirabbit IgG (SA00001-2, 1 : 6000, Proteintech) were then incubated for 90 min. After ECL color exposure, the Odyssey Infrared Imaging System (LI-COR Biosciences, Lincoln, NE, USA) was performed to detect protein bands. *β*-Actin was used as internal references to detect the expression level.

### 2.10. Flow Cytometry

Samples were taken, each about 1 × 10^6^ cells were placed in a 1.5 mL centrifuge tube, the cells were resuspended with 200 *μ*L PBS volume, *α*-SMA (PA5-77846, eBioscience) and s-100 (MA5-32625, eBioscience) antibodies (5 *μ*L for all three antibodies) were added respectively. The aforementioned cells were washed with 1 mL PBS, incubated in the dark for 30 min, and repeated twice. *α*-SMA and s-100 expressions were detected by flow cytometry (A00-1-1102, Beckman) after the cells were resuspended by adding 200 *μ*L PBS, respectively.

### 2.11. Transwell Assay

Cell migration was detected in the Transwell chambers (3428, Corning) with Matrigel Basement Membrane Matrix (354262, BD Biocoat). The cells were digested into single-cell suspension with trypsin and resuspended to 1 × 10^6^/mL in a serum-free medium. About 100 *μ*L cells were added to the upper chamber, and 600 *μ*L of complete medium was added to the lower chamber. The cells were incubated at 37°C for 24 h, wiped with a wet cotton swab, fixed with 4% paraformaldehyde for 20 min, stained with 0.5% crystal violet for 5 to 10 min, observed and photographed under a microscope (Olympus, Japan).

### 2.12. Cell Counting Kit 8 Assay

The cells were inoculated into 96-well plates at the density of 1 × 10^4^ cells/well and incubated in an incubator of 100 *μ*L per well at 37°C and 5% CO_2_. About 10 *μ*L cell counting kit 8 (CCK8; #NU679, Dojindo, Japan) was added to each well for 0, 12, and 24 h after culture. The absorbance (OD) values at 450 nm were analyzed by Bio-Tek microplate (MB-530, Heals, China) after incubation at 37°C and 5% CO_2_ for 4 h.

### 2.13. TUNEL

Cell apoptosis was detected by TUNEL Apoptosis Detection Kit (FITC) (40306ES50, Yelasen, China). Cells in each group were fixed with 3% paraformaldehyde (pH 7.4) at 4°C for 40 min according to the manufacturer's instructions. After washing three times with PBS, 0.1% TritonX-100 and 0.1% sodium citrate solution were added and permerged at 4°C for 5 min. The cells were washed again and treated with fluorescently labeled nucleotides (dUTP) and TdT at 37°C for 60 min. Green fluorescent TUNEL-positive cells were analyzed under a fluorescence microscope.

### 2.14. Targets Prediction and Dual-Luciferase Reporter Verification

The binding sites of lncRNA NEAT1 and miR-132, and miR-132 and MMP9 were predicted using Starbase. To verify the binding of lncRNA NEAT1 with miR-132 and miR-132 with MMP9, wild-type (WT) or mutant (MUT) lncRNA NEAT1 fragments and MMP9 fragments were constructed and inserted into pmirGLO vector (Promega). According to the instructions, the recombinant vector was transfected into the cells using a Lipofectamine 3000 reagent (Thermo Fisher Scientific) and mimics NC and miR-132 mimics were simultaneously transferred into the cells. Finally, the Nano-Glo dual-luciferase reporter assay (Promega) was used to measure luciferase activity.

### 2.15. In Vivo Tumorigenesis

Twenty-four SPF-grade, 4-week-old mice were randomly divided into Model, shNC (HB-derived exosomal shNC), and shNEAT1 (HB-derived exosomal shNEAT1) groups, with 8 mice in each group. Animal studies were approved by the Human Research Ethics Committee of Xiangya Hospital (AF/SQ202104800). Lentiviral vectors of lncRNA NEAT1 were constructed by GeneChem (Shanghai, China) and were transfected into HB cells and then extracted exosomes. For tumor formation, 2 × 10^6^ HB cells were suspended in 200 *μ*L PBS and injected into the subcutaneous area. Exosomes (5 *μ*g) or PBS was then injected intratumorally twice weekly [22]. Tumor volume and weight were measured in each group at 28 d.

### 2.16. Statistical Analysis

Statistical analysis was performed using GraphPad 8.0, and the data of three independent experiments were expressed as mean ± standard deviation (SD). *T*-test was used for data comparison between the two groups, and one-way ANOVA was used for data comparison between multiple groups. *P* < 0.05 was considered statistically significant.

## 3. Results

### 3.1. The Screening of lncRNA NEAT1/miR-132/MMP9

**Figure 1:**
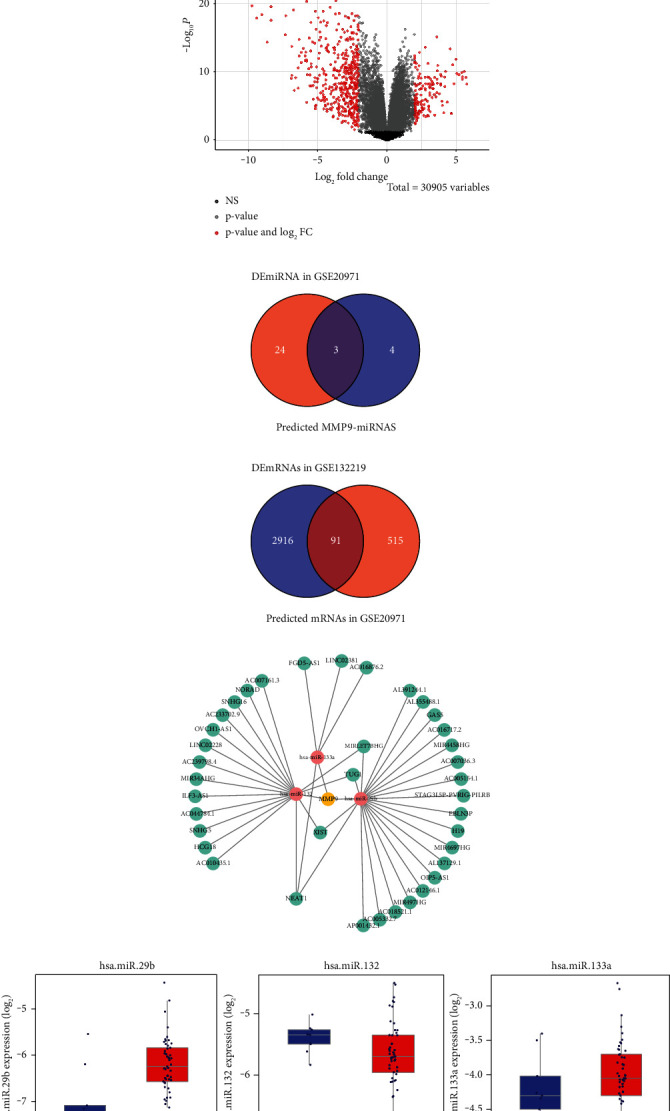
The screening of miRNA: (a) volcano map of differentially expressed miRNAs and mRNAs in HB, (b) volcano map of differentially expressed mRNAs in HB, (c) prediction of MMP9 target miRNAs and differentially expressed miRNAs Venn diagram, (d) prediction of differentially expressed miRNA target genes and differentially expressed mRNAs Venn diagram, (e) differential miRNA-mRNA-lncRNA interaction networks in HB. Among them, orange nodes represented differential mRNAs, red nodes represented miRNAs, and green nodes represented target lncRNAs, (f) box map of hot miRNAs in HB and (g) expression of lncRNA NEAT1 and lncRNA XIST in HB cells was detected by qRT-PCR. ^*∗*^*P* < 0.05 versus the normal liver cells WRL68.

We first analyzed the differential expression of HB-related lncRNAs/miRNAs/mRNAs in HB tissues and paracancerous tissues. The volcano map showed that a total of 606 differentially expressed mRNAs were screened which 466 were down-regulated and 140 were up-regulated. In addition, a total of 27 differentially expressed miRNAs were screened, of which 18 were down-regulated and 9 were up-regulated (Figures 1(a) and 1(b)). The miRNA Venn diagram showed that among the miRNAs targeted by the MMP9 gene and miRNAs with different expressions, there were three differentially expressed miRNAs (Figure 1(c)). As shown in Figure 1(d), there were a total of 91 differentially expressed mRNAs among the mRNAs targeted by differentially expressed miRNAs and the differentially expressed mRNAs.  Figure 1(e) showed the differential miRNA-mRNA-lncRNA interaction network in HB. Among them, orange nodes represented differential mRNAs, red nodes represented miRNAs, and green nodes represented target lncRNAs.  Figure 1(f) showed the box pattern of hot miRNAs in HB. Among them, compared with the normal group, hsa-miR-132 in the tumor group was down-regulated. It was reported that miR-132 could regulate dendritic spines by directly targeting MMP9 mRNA [23]. MMP9 was up-regulated in HB [21]. Therefore, hsa-miR-132 was selected for subsequent studies. We selected normal liver cells and HB cells to verify the expressions of lncRNA NEAT1 and lncRNA XIST by qRT-PCR, and the results showed that lncRNA NEAT1 expression had the most significant difference, and the increase was most obvious in HuH-6 cells (Figure 1(g)). Therefore, we chose lncRNA NEAT1 and HuH-6 cells for the follow-up study. So we focused on the regulatory mechanism of lncRNA NEAT1/miR-132/MMP9 in HB.

### 3.2. lncRNA NEAT1 and MMP9 Expressions Were Increased in Tumor Tissues, While miR-132 Expression Was Decreased

**Figure 2:**
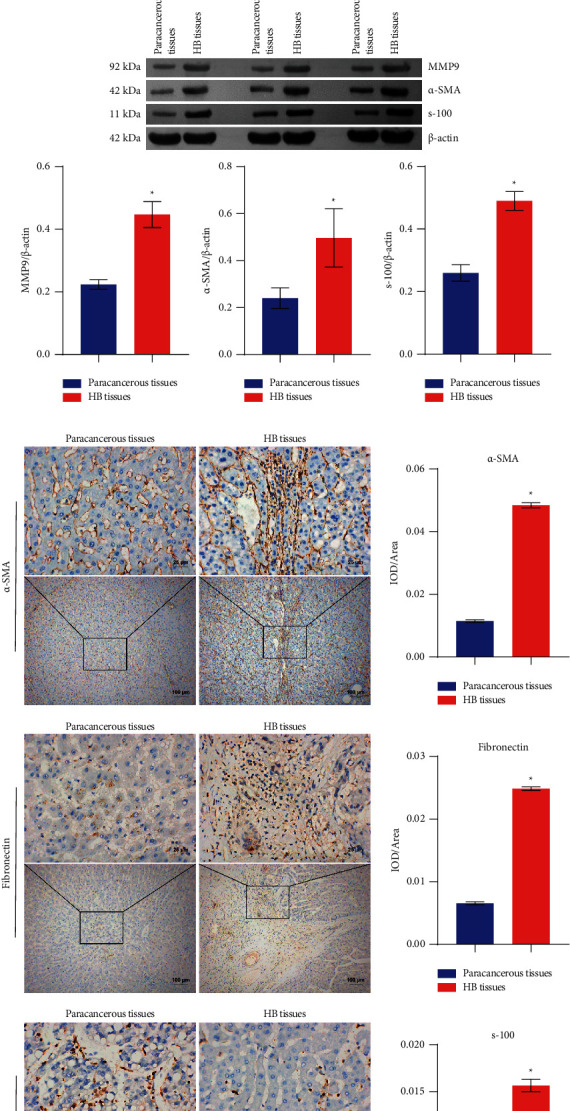
LncRNA NEAT1 and MMP9 expressions were increased in HB tissues, while miR-132 expression was decreased: (a) the expression of lncRNA NEAT1, miR-132, and MMP9 was characterized by qRT-PCR, (b) western blot was used to detect MMP9, *α*-SMA, and s-100 expressions, and (c) *α*-SMA, fibronectin, and s-100 expressions in HB tissues and paracancerous tissues were detected by IHC (×400, 25 *μ*m; ×100, 100 *μ*m). ^*∗*^*P* < 0.05 versus paracancerous tissues.

To verify lncRNA NEAT1, miR-132, and MMP9 expressions, HB tissues and paracancerous tissues were first taken and qRT-PCR was performed to detect lncRNA NEAT1, miR-132, and MMP9 expressions. The results showed that lncRNA NEAT1 and MMP9 expressions were increased in HB tissues, while miR-132 expression was decreased compared with paracancerous tissues (Figure 2(a)). Western blot results showed that MMP9, *α*-SMA, and s-100 expressions were all increased in HB tissues compared with those in paracancerous tissues (Figure 2(b)). The whole blots used in this manuscript were shown in Supplementary file 1. To detect fibrosis in HB tissues and paracancerous tissues, IHC was used to detect *α*-SMA, fibronectin, and s-100 expressions. As shown in Figure 2(c), *α*-SMA, fibronectin, and s-100 were all positively expressed in HB tissues compared with paracancerous tissues. This indicated the presence of fibrosis in the HB tissues.

### 3.3. HB-Derived Exosomal lncRNA NEAT1 Regulated miR-132 and MMP9 Expression in BMSCs

**Figure 3:**
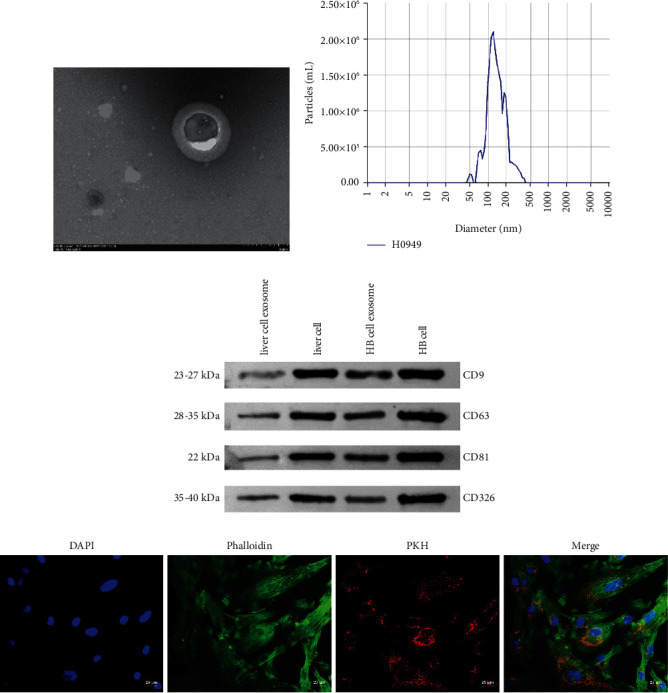
HB-derived exosomal lncRNA NEAT1 regulated miR-132 and MMP9 expression in BMSCs: (a) TEM was used to characterize the morphology of exosomes, (b) NTA was used to analyze exosomes size, (c) Western blot characterization of CD9, CD63, CD81, CD326 exosome marker proteins, and (d) exosome uptake assay (×400, 25 *μ*m). ^*∗*^*P* < 0.05.

**Figure 4:**
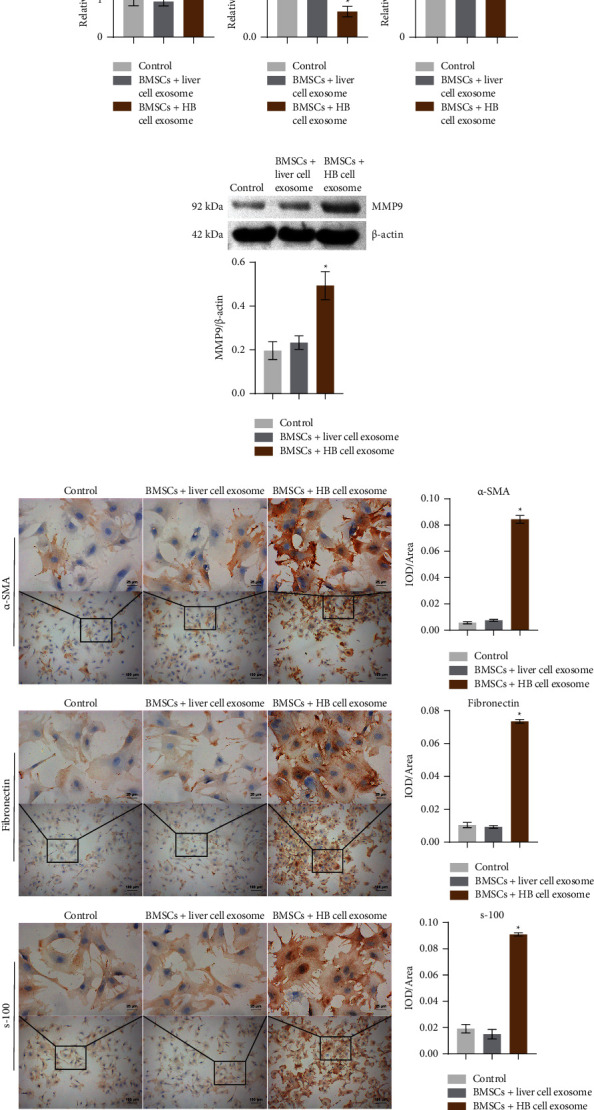
HB-derived exosomal lncRNA NEAT1 regulated miR-132 and MMP9 expression in BMSCs: (a) expression of lncRNA NEAT1, miR-132, and MMP9 was detected by qRT-PCR, (b) expression of MMP9 was measured by Western blot, and (c) ICC was applied to detect *α*-SMA, fibronectin, and s-100 expressions in BMSCs (×400, 25 *μ*m; ×100, 100 *μ*m). ^*∗*^*P* < 0.05 versus BMSC + liver cell exosome.

Exosomes play an essential role in the occurrence and evolution of HB, including promoting the formation of the liver cancer microenvironment, enhancing tumor invasion and metastasis, enhancing angiogenesis, and participating in tumor immunosuppression and tumor chemoradiotherapy resistance [12, 13]. In order to identify exosomes extracted from HB cells and normal liver cells, it was observed under TEM that exosomes of HB cells and normal liver cells had round and oval vesicle-like structures and the size of exosomes was 100 to 200 nm (Figures 3(a) and 3(b)). In addition, Western blot analysis showed that liver cell exosome and HB cell exosome expressed CD9, CD63, CD81, and CD326 (Figure 3(c)). As shown in Figure 3(d), PKH67 labeled exosomes were co-cultured with BMSCs, and phalloidin stained the cytoskeleton to show that BMSCs could absorb exosomes. LncRNA NEAT1, miR-132, and MMP9 expressions were further characterized by qRT-PCR or Western blot. Compared with the BMSC + liver cell exosome group, miR-132 expression was decreased while lncRNA NEAT1 and MMP9 expression was increased in the BMSC + HB cell exosome group (Figures 4(a) and 4(b)). ICC results showed that compared with the BMSC + liver cell exosome group, *α*-SMA, fibronectin, and s-100 were positive in the BMSC + HB cell exosome group (Figure 4(b)). These results indicated that HB-derived exosomal lncRNA NEAT1 regulated miR-132 and MMP9 expression in BMSCs.

### 3.4. lncRNA NEAT1 Regulated MMP9 through miR-132

**Figure 5:**
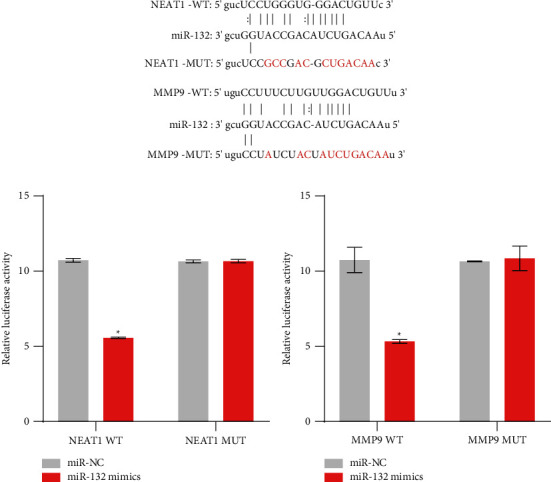
LncRNA NEAT1 regulated MMP9 through miR-132: (a) bioinformatics predicted the targets of lncRNA NEAT1 and miR-132, and miR-132 and MMP9 and (b) dual-luciferase reporter assay was applied to demonstrate the interaction between lncRNA NEAT1 and miR-132, and miR-132 and MMP9. ^*∗*^*P* < 0.05 versus miR-NC.

We found that lncRNA NEAT1, miR-132, and MMP9 played an important role in HB through the aforementioned study. To further explore whether it played a role through lncRNA NEAT1/miR-132/MMP9 axis, we used bioinformatics to predict lncRNA NEAT1 and miR-132 and miR-132 and MMP9 binding sites. As shown in Figure 5(a), lncRNA NEAT1 had binding sites with miR-132, and miR-132 also had binding sites with MMP9. Dual-luciferase reporter assay results showed that compared with miR-NC, the luciferase activity of lncRNA NEAT1 WT was decreased with the addition of miR-132 mimics, while the luciferase activity of lncRNA NEAT1 Mut remained unchanged. It was shown that lncRNA NEAT1 could bind to miR-132 (Figure 5(b)). In addition, compared with miR-NC, the luciferase activity of MMP9 WT decreased after the addition of miR-132 mimics, while the luciferase activity of MMP9 Mut remained basically unchanged, indicating that miR-132 could bind to MMP9 (Figure 5(b)). These results suggested that lncRNA NEAT1 regulated MMP9 through miR-132.

### 3.5. HB-Derived Exosomal lncRNA NEAT1 Promoted BMSCs Differentiation toward Invasive Myofibroblasts via miR-132/MMP9 Axis

**Figure 6:**
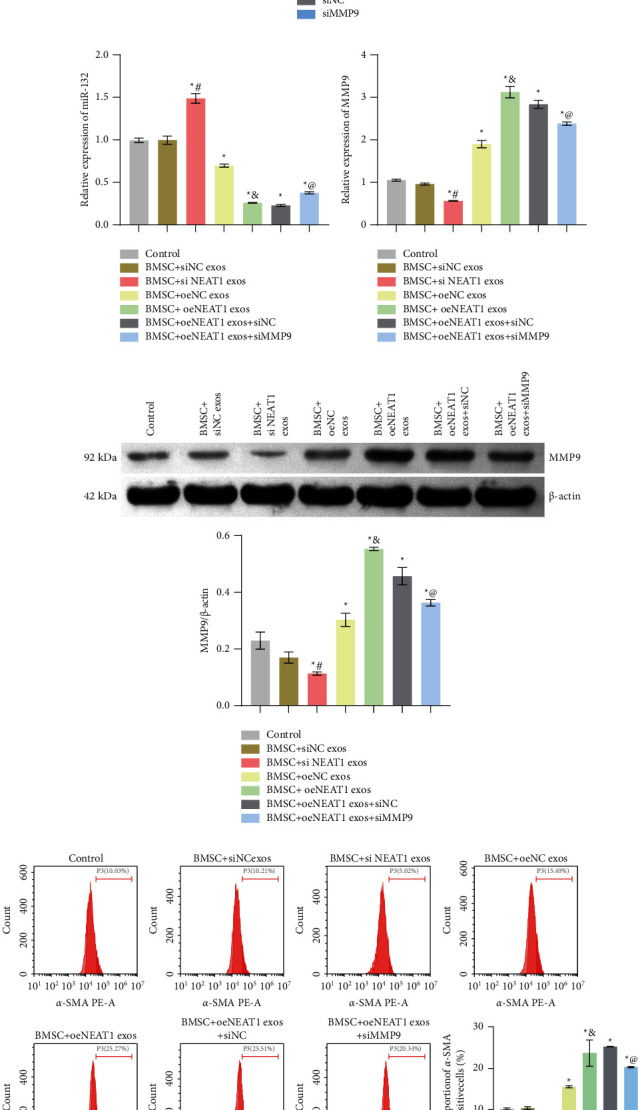
HB-derived exosomal lncRNA NEAT1 promoted BMSCs differentiation toward invasive myofibroblasts via miR-132/MMP9 axis: (a) the knockdown efficiency of lncRNA NEAT1 was detected by qRT-PCR, ^*∗*^*P* < 0.05, (b) the overexpression efficiency of lncRNA NEAT1 was measured by qRT-PCR, ^*∗*^*P* < 0.05, (c) the knockdown efficiency of MMP9 was detected by qRT-PCR, ^*∗*^*P* < 0.05, (d) expression of miR-132 and MMP9 was characterized by qRT-PCR, (e) expression of MMP9 was characterized by Western blot, and (f) flow cytometry was used to detect *α*-SMA and s-100 expressions, ^*∗*^*P* < 0.05 versus control, ^#^*P* < 0.05 versus BMSC + siNC exos, ^&^*P* < 0.05 versus BMSC + oeNC exos, ^@^*P* < 0.05 versus BMSC + oeNEAT1 exos + siNC.

**Figure 7:**
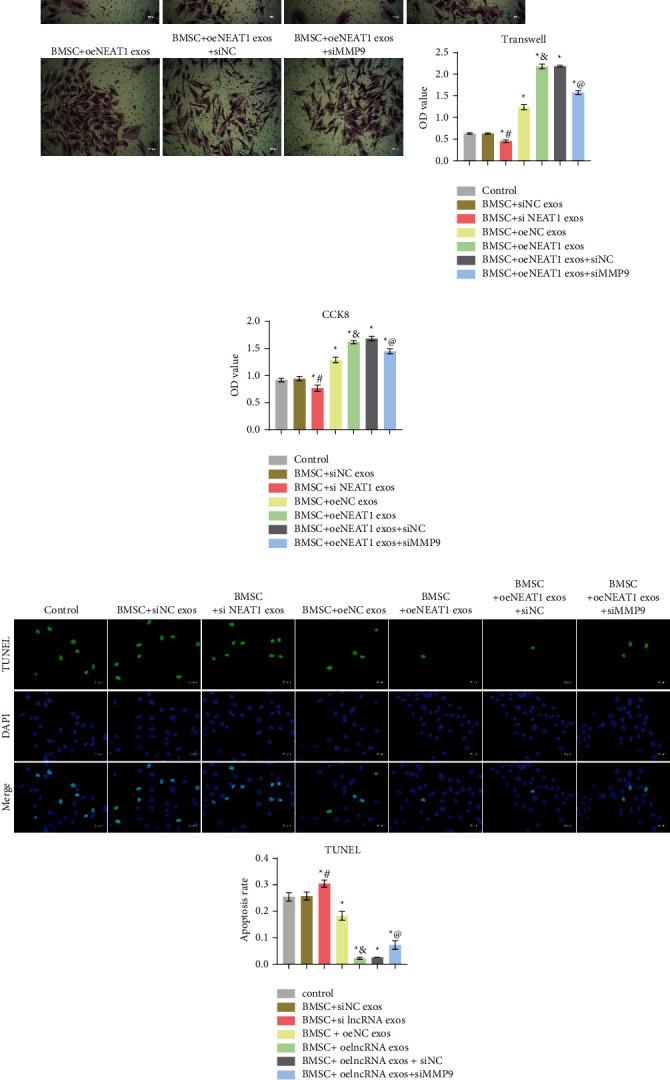
HB-derived exosomal lncRNA NEAT1 promoted BMSCs differentiation toward invasive myofibroblasts via miR-132/MMP9 axis: (a) transwell assay was used to measure cell migration ability (×100, 100 *μ*m), (b) cell proliferation ability was detected by CCK-8 assay, (c) TUNEL fluorescence detection of cell apoptosis (×400, 25 *μ*m). ^*∗*^*P* < 0.05 versus Control, ^#^*P* < 0.05 versus BMSC + siNC exos, ^&^*P* < 0.05 versus BMSC + oeNC exos, ^@^*P* < 0.05 versus BMSC + oeNEAT1 exos + siNC.

To investigate whether lncRNA NEAT1 affected BMSCs differentiation to invasive myofibroblasts through miR-132/MMP9 axis, we performed knockdown and overexpression of lncRNA NEAT1. At the same time, we knocked down MMP9. Firstly, we detected the knockdown and overexpression efficiency of lncRNA NEAT1. As shown in Figure 6(a), compared with the siNC group, the expression of lncRNA NEAT1 in the siNEAT1 group was decreased. Compared with the siNC exos group, the expression of lncRNA NEAT1 in the siNEAT1 exos group was also decreased. This showed that we successfully knocked down lncRNA NEAT1. Compared with the oeNC group, the expression of lncRNA NEAT1 in the oeNEAT1 group was increased. The expression of lncRNA NEAT1 in the oeNEAT1 exos group also increased than that in the oeNC exos group (Figure 6(b)). This showed that we have successfully overexpressed lncRNA NEAT1. Furthermore, as shown in Figure 6(c), compared with the siNC group, the expression of MMP9 in the siMMP9 group was reduced. This showed that we successfully knocked down MMP9. Then, miR-132 and MMP9 expressions were characterized by qRT-PCR or Western blot. The results showed that miR-132 expression was increased, and MMP9 expression was decreased in the BMSC + siNEAT1 exos group. Compared with the BMSC + oeNC exos group, miR-132 expression was decreased and MMP9 expression was increased in the BMSC + oeNEAT1 exos group. In addition, miR-132 expression was increased and MMP9 expression was reduced in the BMSC + oeNEAT1 exos + siMMP9 group compared with the BMSC + oeNEAT1 exos + siNC group (Figures 6(d) and 6(e)). Flow cytometry staining for *α*-SMA and s-100 results showed that the positive expression of *α*-SMA and s-100 decreased in the BMSC + siNEAT1 exos group. Compared with the BMSC + oeNC exos group, the positive expression of *α*-SMA and s-100 increased in the BMSC + oeNEAT1 exos group. Furthermore, the positive expression of *α*-SMA and s-100 was decreased in the BMSC + oeNEAT1 exos + siMMP9 group compared with the BMSC + oeNEAT1 exos + siNC group (Figure 6(f)). Cell function experiment results were shown in Figures 7(a)to 7(c). The BMSC + siNEAT1 exos group showed decreased migration and proliferation ability and increased apoptosis. Compared with the BMSC + oeNC exos group, the BMSC + oeNEAT1 exos group showed increased ability of migration and proliferation and reduced apoptosis, while the BMSC + oeNEAT1 exos + siMMP9 group showed the decreased ability of migration and proliferation, and increased apoptosis compared with the BMSC + oeNEAT1 exos + siNC group. These results indicated that HB-derived exosomal lncRNA NEAT1 promoted BMSCs differentiation toward invasive myofibroblasts via miR-132/MMP9 axis.

### 3.6. HB-Derived Exosomal lncRNA NEAT1 Affected the Development of HB

**Figure 8:**
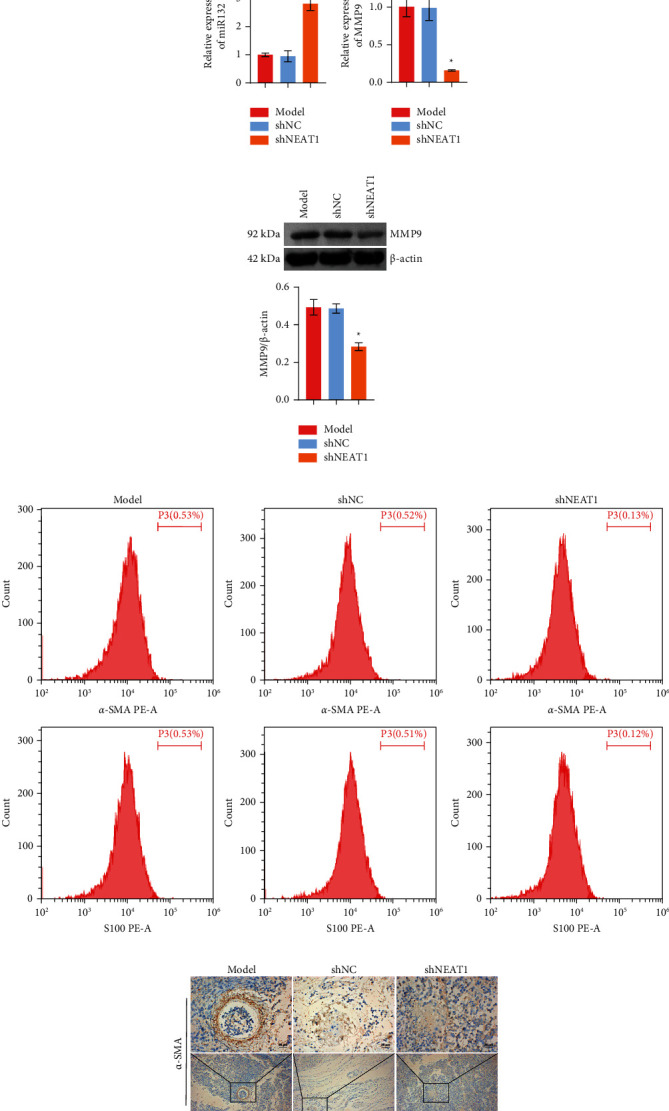
HB-derived exosomal lncRNA NEAT1 affected the development of HB: (a–c) Characterization of tumor size, volume, and weight, (d) the expression of lncRNA NEAT1 was detected by qRT-PCR, (e) the expression of miR-132 was measured by qRT-PCR, (f–g) qRT-PCR and Western blot were used to detect MMP9 expression, respectively, (h) flow cytometry was performed to detect *α*-SMA and s-100 expressions, and (i) *α*-SMA, fibronectin, and s-100 expressions were detected by IHC (×400, 25 *μ*m; ×100, 100 *μ*m). ^*∗*^*P* < 0.05 versus shNC.

In order to investigate the development of HB in vivo, we knocked down the lncRNA NEAT1 and carried out tumorigenesis experiments in nude mice.  Figure 8(a) shows the size of the tumor. Compared with the shNC group, the tumor size of shNEAT1 group was reduced. In addition, compared with the shNC group, the volume, and weight of shNEAT1 group was also decreased (Figures 8(b) and 8(c)). As shown in Figures 8(d) and 8(e), compared with the shNC group, lncRNA NEAT1 expression was decreased in the shNEAT1 group, while miR-132 expression was increased. qRT-PCR and Western blot results showed that MMP9 expression was decreased in the shNEAT1 group compared with the shNC group (Figures 8(f) and 8(g)). Flow cytometry staining for *α*-SMA and s-100 showed the positive expression of *α*-SMA and s-100 was decreased in the shNEAT1 group compared with the shNC group (Figure 8(h)). IHC results showed that compared with the shNC group, the positive expressions of *α*-SMA, fibronectin, and s-100 were decreased in the shNEAT1 group (Figure 8(i)). These results indicated that HB-derived exosomal lncRNA NEAT1 could affect the development of HB in vivo.

## 4. Discussion

HB is the most common liver malignancy in children. Currently, there is a lack of effective and low-cost treatment. In this study, we screened the differentially expressed lncRNA NEAT1/miR-132/MMP9 based on bioinformatics analysis and cell verification. Based on this, we conducted a number of experiments to show that HB-derived exosomal lncRNA NEAT1 induced BMSCs to differentiate into tumor-supporting myofibroblasts by regulating the miR-132/MMP9 axis.

At present, the difference in exosomes secreted by normal cells and cancer cells has been reported. For example, Clark et al. pointed out in the study of nonsmall-cell lung cancer (NSCLC) that compared with the immortalized normal epithelial cell-derived exosomes, proteins enriched in the NSCLC exosomal proteome include proteins associated with cell invasion, angiogenesis, and cell proliferation, and NSCLC exosomes can actively regulate the proliferative capacity of recipient cells [24]. Stefanius et al. reported that human pancreatic cancer cell exosomes, but not human normal cell exosomes, act as an initiator in cell transformation [25]. Singh et al. showed that treatment with exosomes derived from metastatic breast cancer MDA-MB-231 cells, including the highly-expressed miR-10b when compared with nonmetastatic breast cancer MCF-7 cells or nonmalignant breast HMLE or MCF-10A cells, was also observed to induce the invasive ability of nonmalignant mammary epithelial cells [26]. These studies indicated that cancer cells derived exosomes may represent a more effective approach for disease treatment. In addition, cancer cell-derived exosomes play an important role in cancer progression and the regulation of tumor microenvironment [27]. Cancer-derived exosomes have great potential to be biomarkers for early clinical diagnosis and evaluation of the efficacy of cancer treatment [28]. In various tumor types, lncRNAs carrying exosomes are actively released from tumor cells [29]. LncRNA NEAT1 is one of the most widely studied lncRNAs associated with a variety of human cancers. LncRNA NEAT1 could regulate proliferation, apoptosis, and invasion of liver cancer [30]. LncRNA NEAT1 also could promote the progression of colorectal cancer by activating Wnt/*β*-catenin signaling through interaction with DDX5 [31]. In addition, lncRNA NEAT1 could promote SOX2 expression by inhibiting miR-132, thus promoting glioma development [32]. These reports suggested that lncRNA NEAT1 can bind to miR-132 and thus play a role in disease. miR-132 has been reported to be down-regulated in several human cancers and is associated with tumor progression [33]. It was reported that miR-132 could regulate dendritic spines by directly targeting MMP9 mRNA [23]. However, there are few studies on miR-132/MMP-9 in HB. In this study, we screened lncRNA NEAT1/miR-132/MMP9 through bioinformatics analysis. In clinical samples, lncRNA NEAT1 and MMP9 expressions were elevated in tumor tissues, while miR-132 expression was decreased. Bioinformatics prediction and dual-luciferase reporter assay demonstrated that lncRNA NEAT1 regulated MMP9 through miR-132. These further proved that lncRNA NEAT1/miR-132/MMP9 axis might play a regulatory role in HB.

MSCs have been shown to be able to differentiate into cells conducive to tumor growth and invasion. Under the induction of tumor microenvironment, BMSCs can differentiate into myofibroblasts, thereby affecting the remodeling of tumor mesenchymal microenvironment [34]. The tumor-tracking properties of MSCs provide an attractive opportunity to target transgenic transfer to tumor-forming sites [35]. It has been reported that exosomes of human hepatocellular carcinoma HepG2 cells can induce differentiation of human adipose derived MSCs into cancer-associated myofibroblasts [36]. Colorectal cancer exosomes can induce morphological and functional changes in colorectal MSCs and actively involve tumor growth and malignant progression [37]. However, few studies on HB-derived exosomes have been reported. In our research, we found that HB-derived exosomal lncRNA NEAT1 regulated miR-132 and MMP9 expression in BMSCs. Myofibroblasts are key components of tumor stroma and secrete paracrine factors to support rapid tumor growth and metastasis, leading to tumor invasion and poor prognosis [38]. It has been reported that breast cancer-derived exosomes activate cancer-related fibroblasts in the tumor microenvironment through miR-146a to regulate the invasion and metastasis of breast cancer cells [39]. However, there are few studies on cancer-derived exosomal lncRNA in HB. Our results revealed that HB-derived exosomal lncRNA NEAT1 promoted BMSCs differentiation toward invasive myofibroblasts via miR-132/MMP9 axis. Tumor formation experiments in nude mice showed that HB-derived exosomal lncRNA NEAT1 could affect the development of HB.

However, there are some limitations to the study. In this paper, due to financial constraints, only one cell line was used in the study of the mechanism. In the future, we will use two or more cell lines to conduct in-depth study of the role of lncRNA NEAT1/miR-132/MMP9 in HB and the mechanisms involved.

In conclusion, our results suggested that HB-derived exosomal lncRNA NEAT1 induced MSCs to differentiate into tumor-supporting myofibroblasts by regulating the miR-132/MMP9 axis, which provided a reference and basis for clinical treatment and prognosis judgment of HB in the future and also provided a new target for the treatment of HB.

## Data Availability

The data used to support the findings of this study are available from the corresponding author upon request.

## Supplementary Materials

Supplementary Materials
*Supplementary File 1.* Whole western blot bands for all figures.
