# Supplementary material for: Hepatoblastoma: Derived Exosomal LncRNA NEAT1 Induces BMSCs Differentiation into Tumor-Supporting Myofibroblasts via Modulating the miR-132/MMP9 Axis
Source: J Oncol. 2022 Mar 8;2022:7630698. doi: 10.1155/2022/7630698 (PMC8923764; doi:10.1155/2022/7630698)

Fig.2B: MMP9

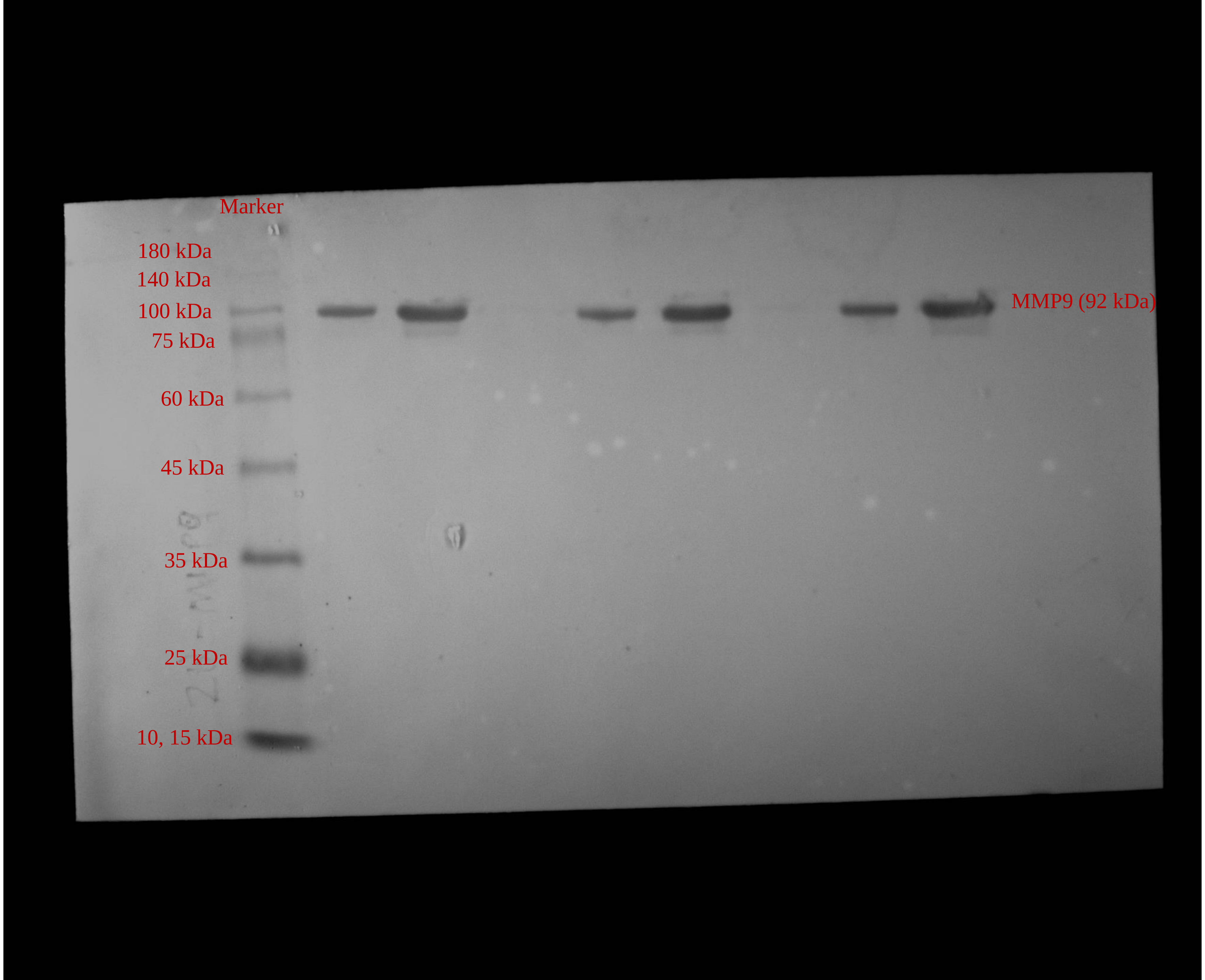
Marker
180 kDa
140 kDa
100 kDa
75 kDa
60 kDa
45 kDa
35 kDa
25 kDa
10, 15 kDa
MMP9 (92 kDa)

Fig.2B: s-100

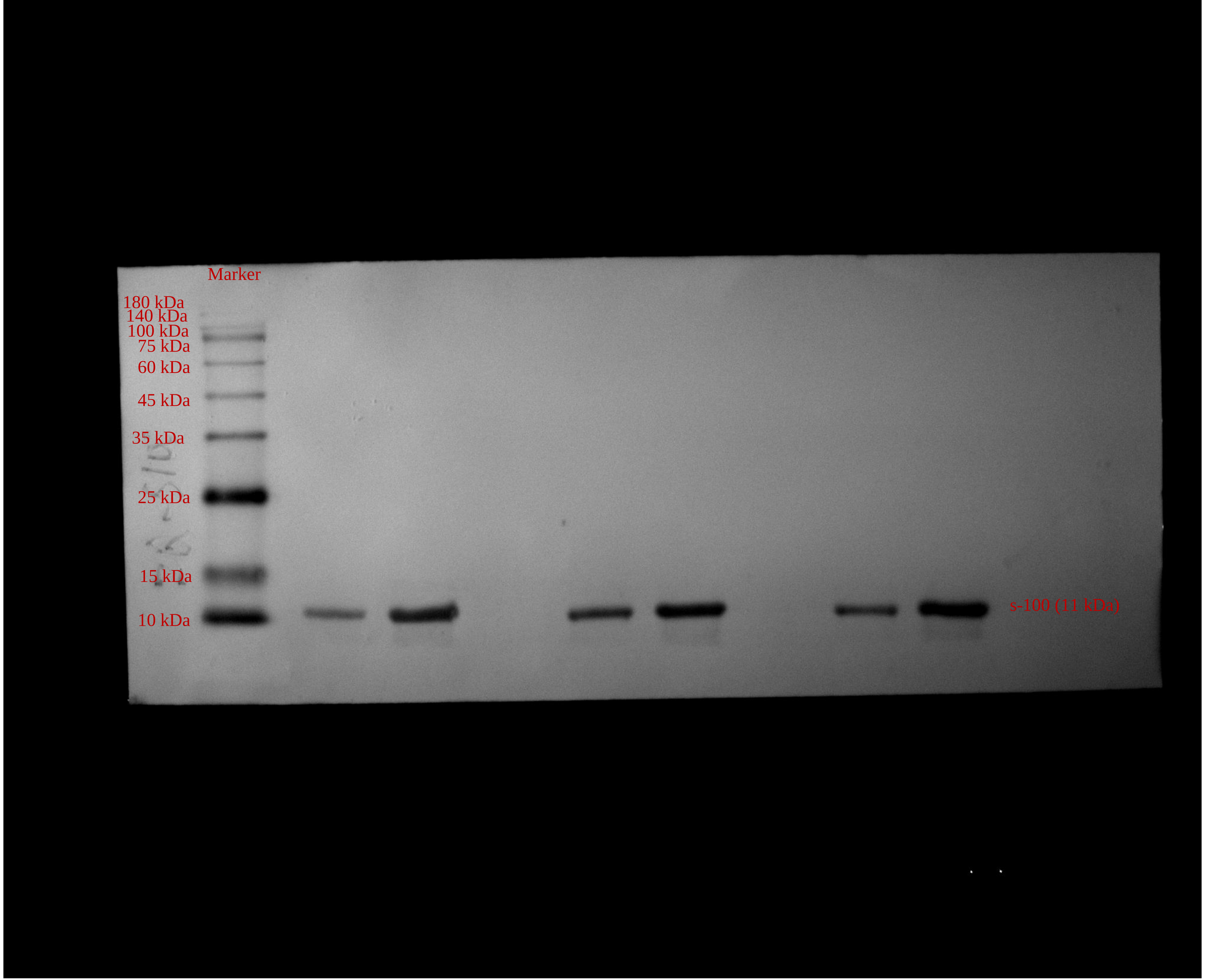
Marker
180 kDa
140 kDa
100 kDa
75 kDa
60 kDa
45 kDa
35 kDa
25 kDa
15 kDa
10 kDa
s-100 (11 kDa)

Fig.2B: α-SMA

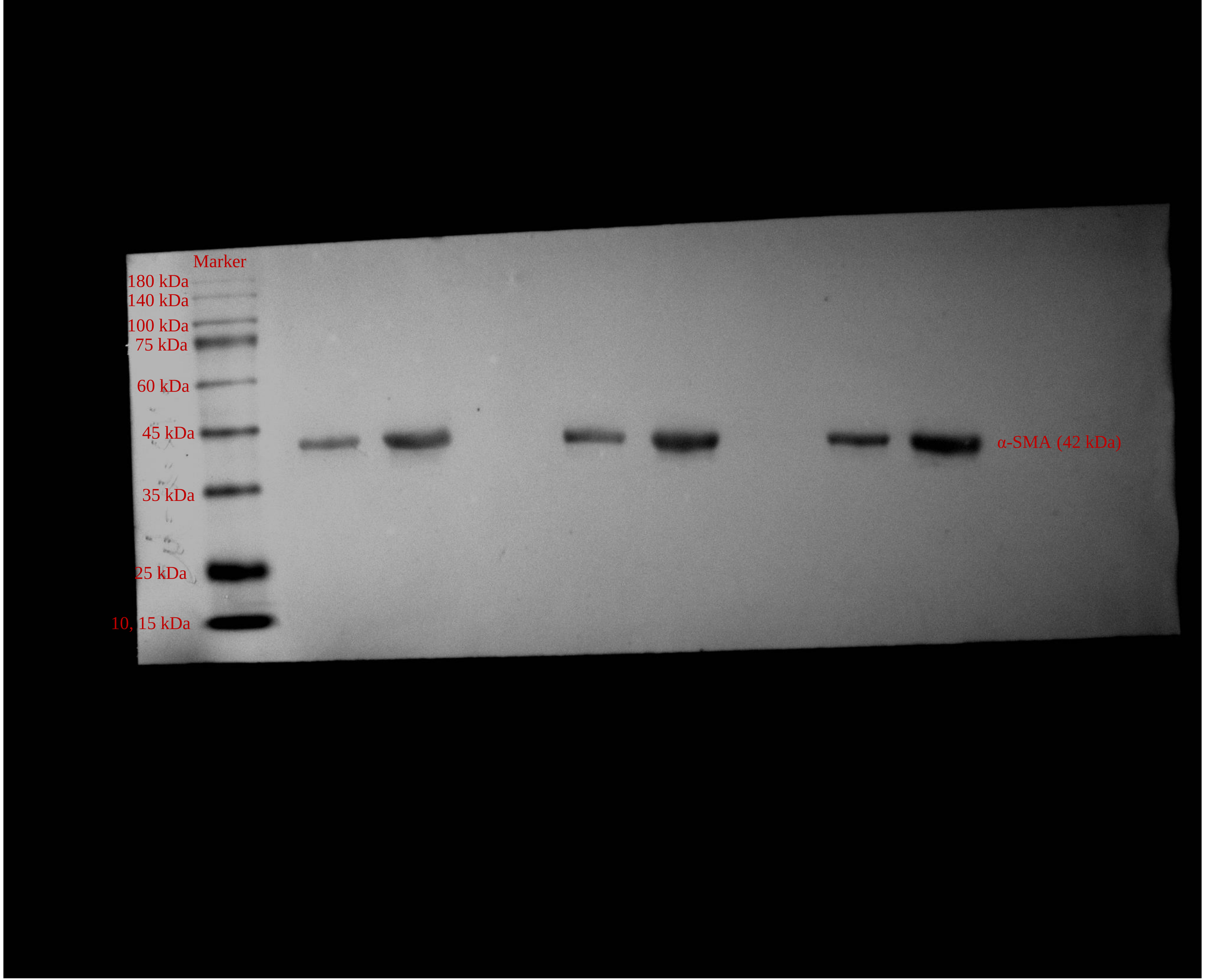

Marker
180 kDa
140 kDa
100 kDa
75 kDa
60 kDa
45 kDa
35 kDa
25 kDa
10, 15 kDa
α-SMA (42 kDa)

Fig.2B: β-actin

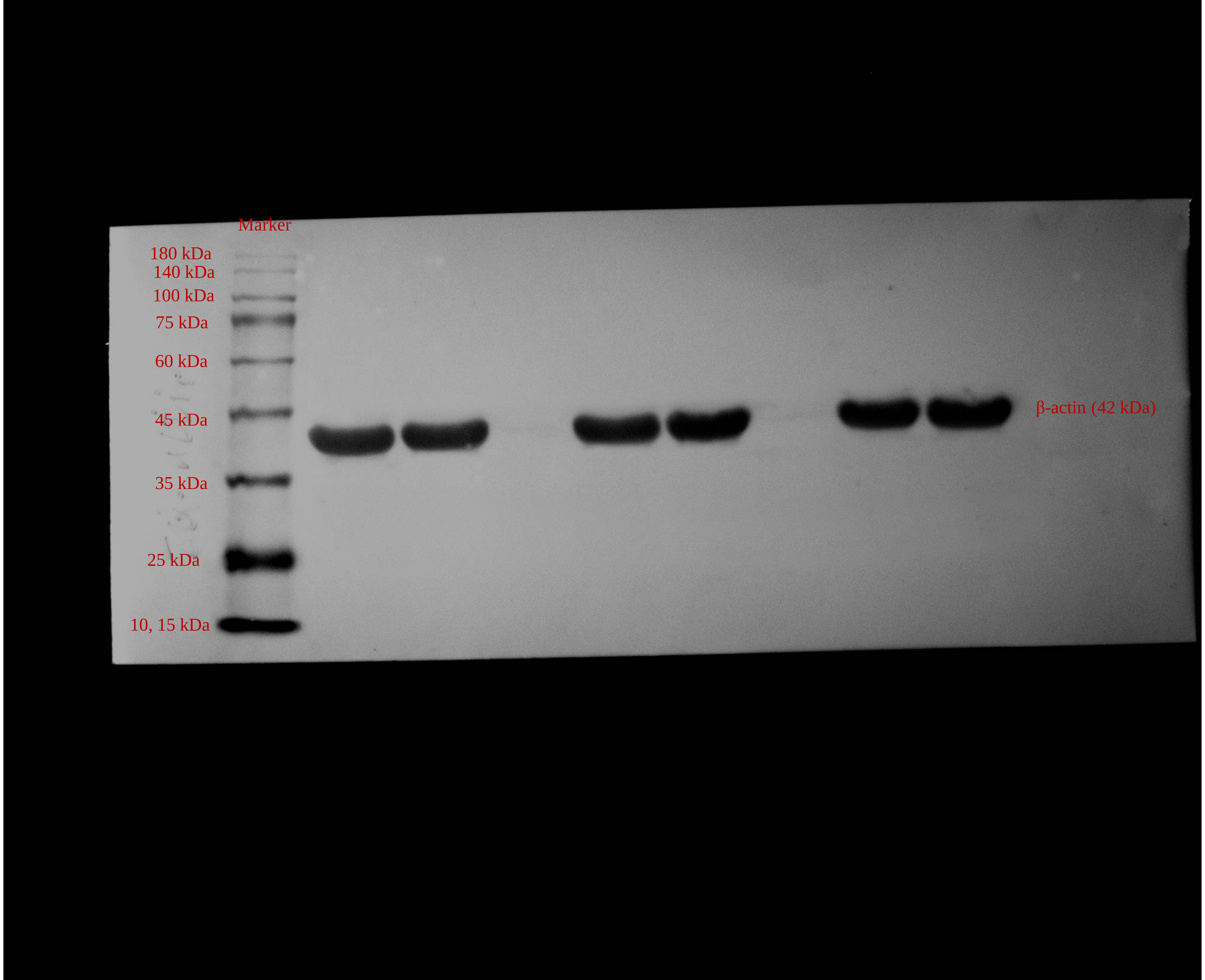

Fig.3C: CD9

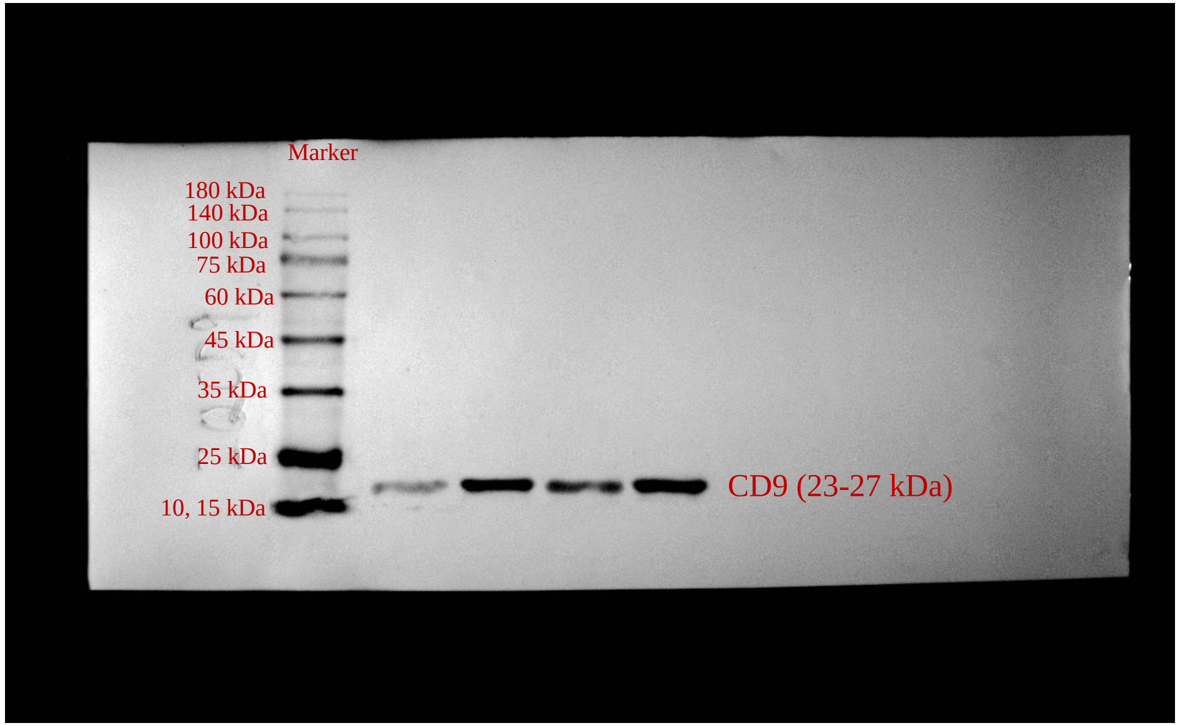

Marker
180 kDa
140 kDa
100 kDa
75 kDa
60 kDa
45 kDa
35 kDa
25 kDa
10, 15 kDa
CD9 (23-27 kDa)

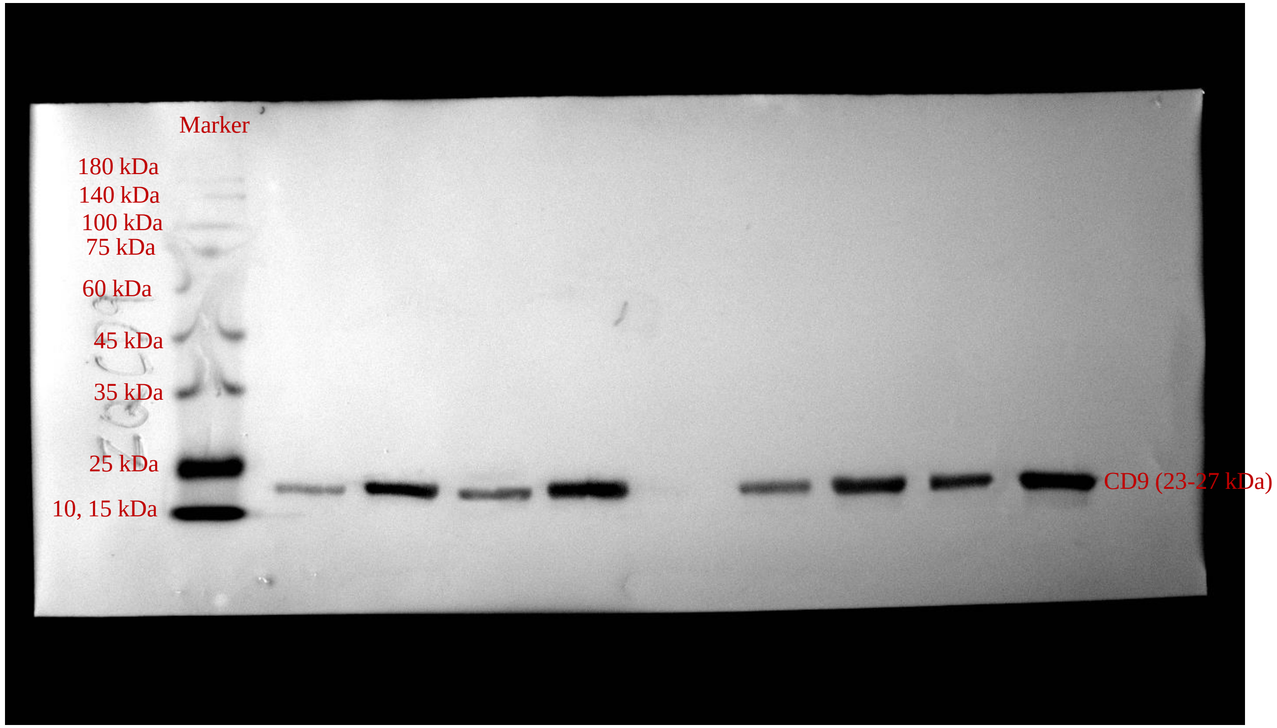

Marker
180 kDa
140 kDa
100 kDa
75 kDa
60 kDa
45 kDa
35 kDa
25 kDa
10, 15 kDa
CD9 (23-27 kDa)

Fig.3C: CD63

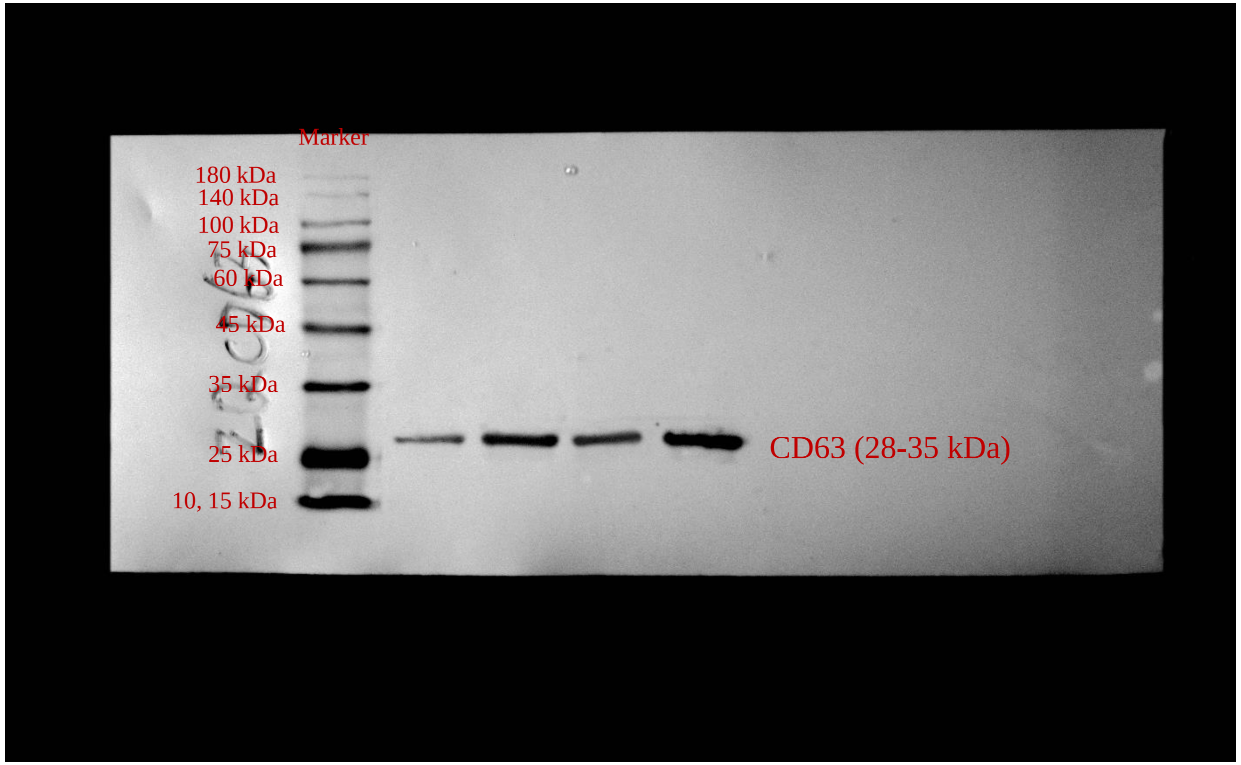

Marker
180 kDa
140 kDa
100 kDa
75 kDa
60 kDa
45 kDa
35 kDa
25 kDa
10, 15 kDa
CD63 (28-35 kDa)

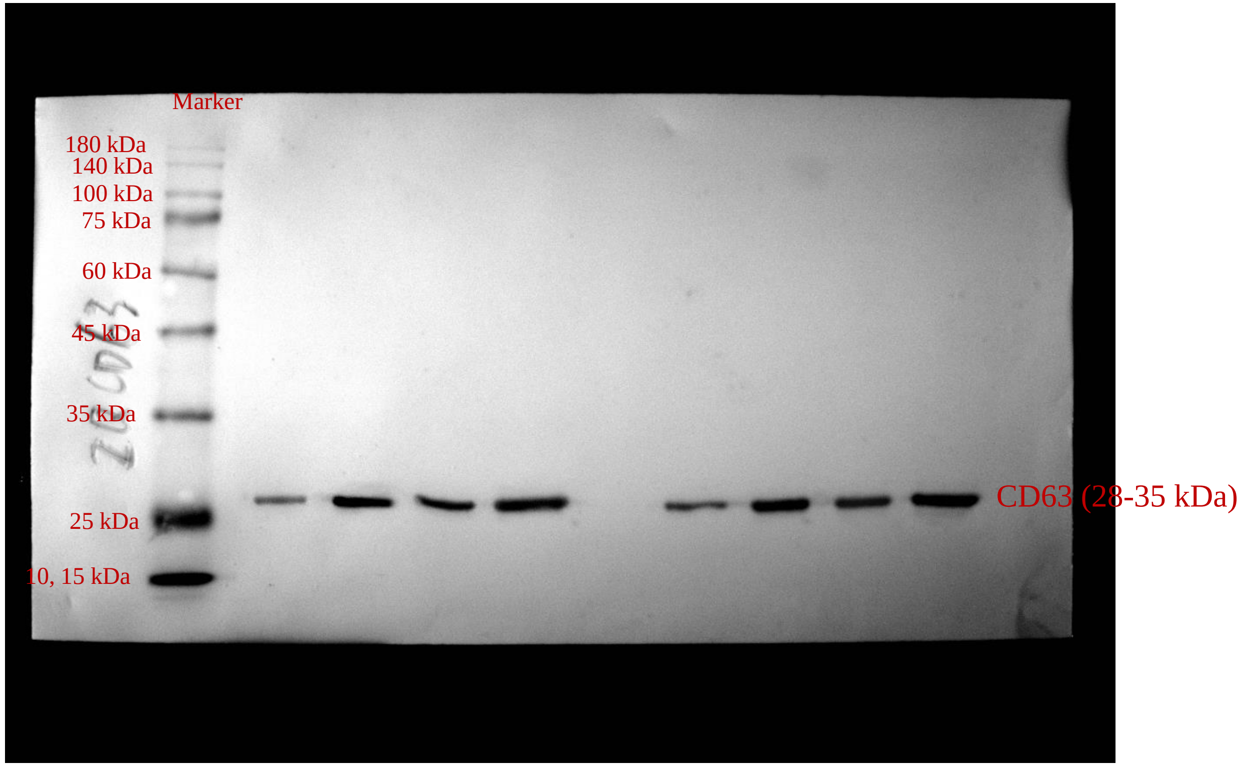

Marker
180 kDa
140 kDa
100 kDa
75 kDa
60 kDa
45 kDa
35 kDa
25 kDa
10, 15 kDa
CD63 (28-35 kDa)

Fig.3C: CD81

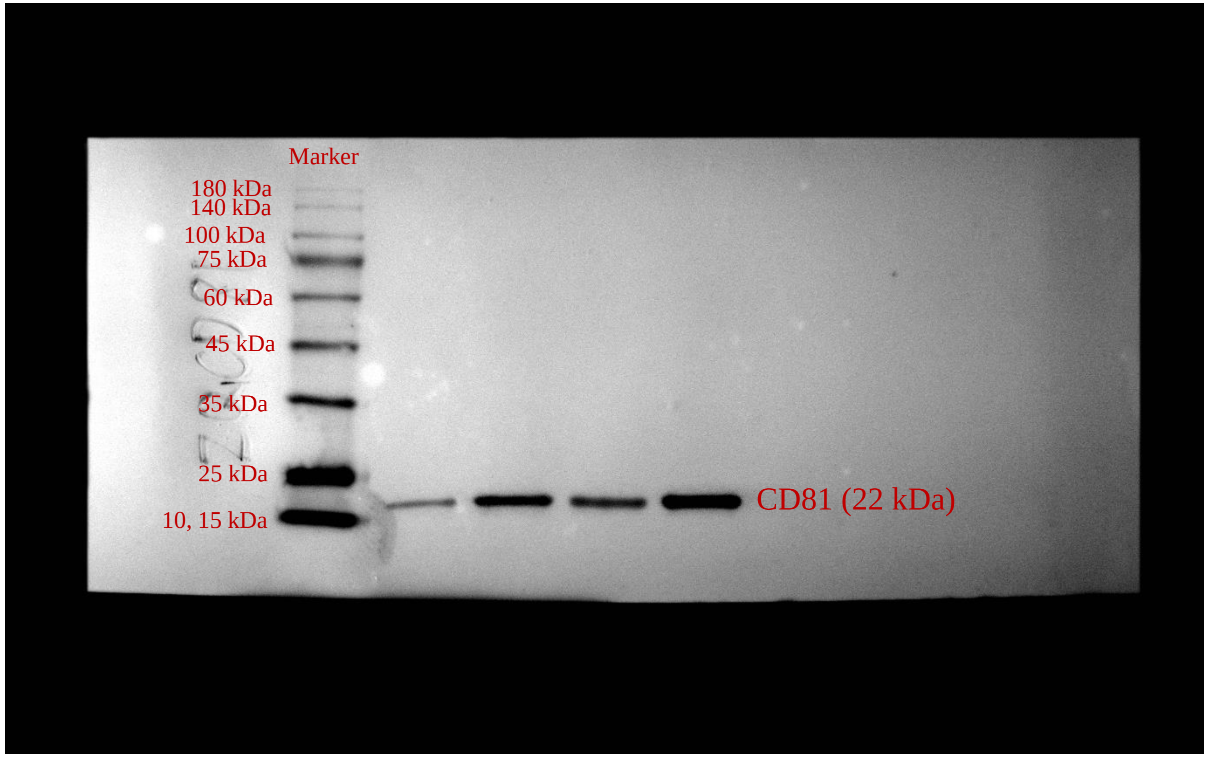

Marker
180 kDa
140 kDa
100 kDa
75 kDa
60 kDa
45 kDa
35 kDa
25 kDa
10, 15 kDa
CD81 (22 kDa)

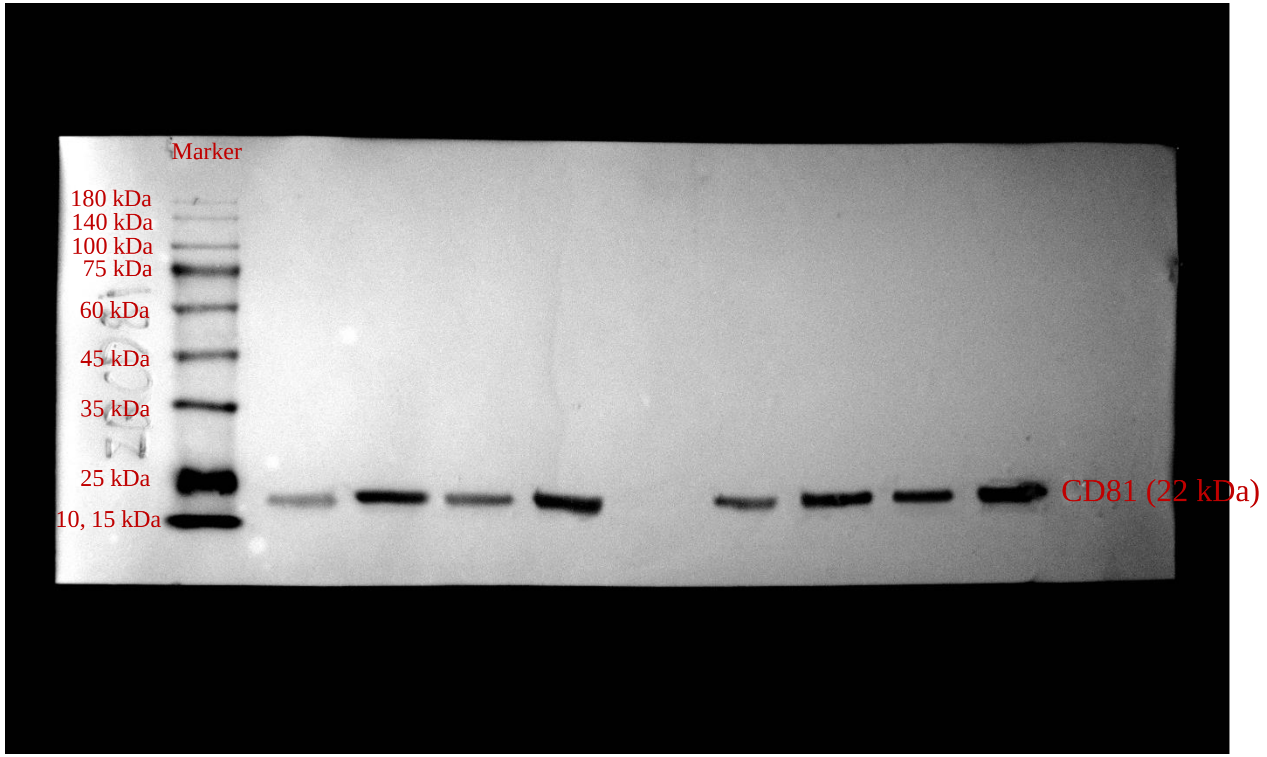

Marker
180 kDa
140 kDa
100 kDa
75 kDa
60 kDa
45 kDa
35 kDa
25 kDa
10, 15 kDa
CD81 (22 kDa)

Fig.3C: CD326

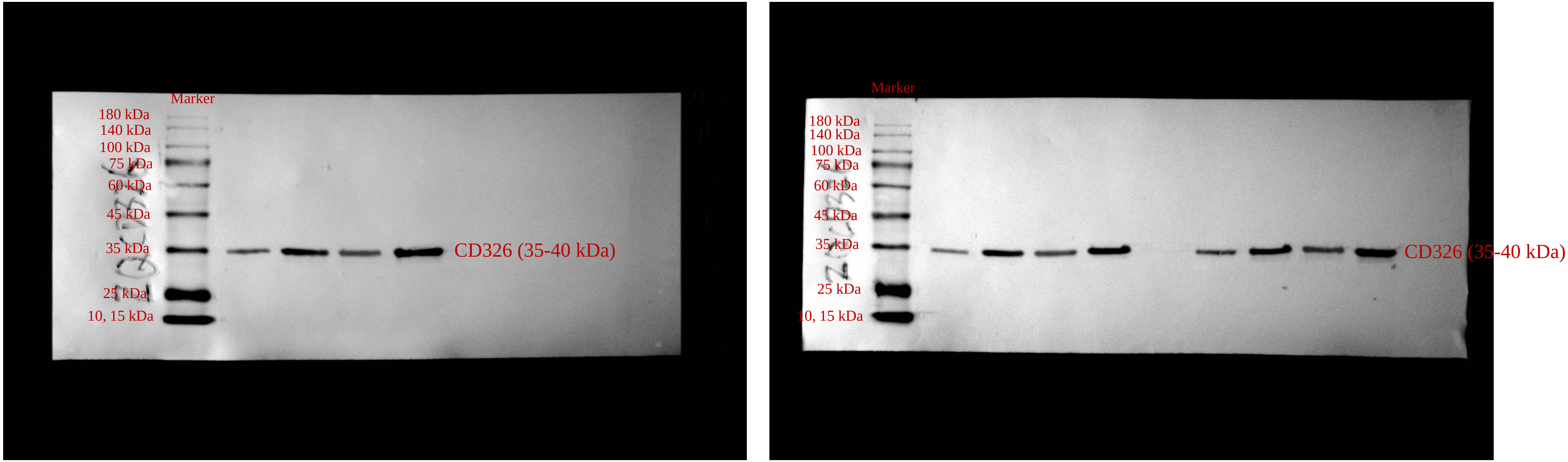
Marker
180 kDa
140 kDa
100 kDa
75 kDa
60 kDa
45 kDa
35 kDa
25 kDa
10, 15 kDa
CD326 (35-40 kDa)
Marker
180 kDa
140 kDa
100 kDa
75 kDa
60 kDa
45 kDa
35 kDa
25 kDa
10, 15 kDa
CD326 (35-40 kDa)

Fig.4B: MMP9

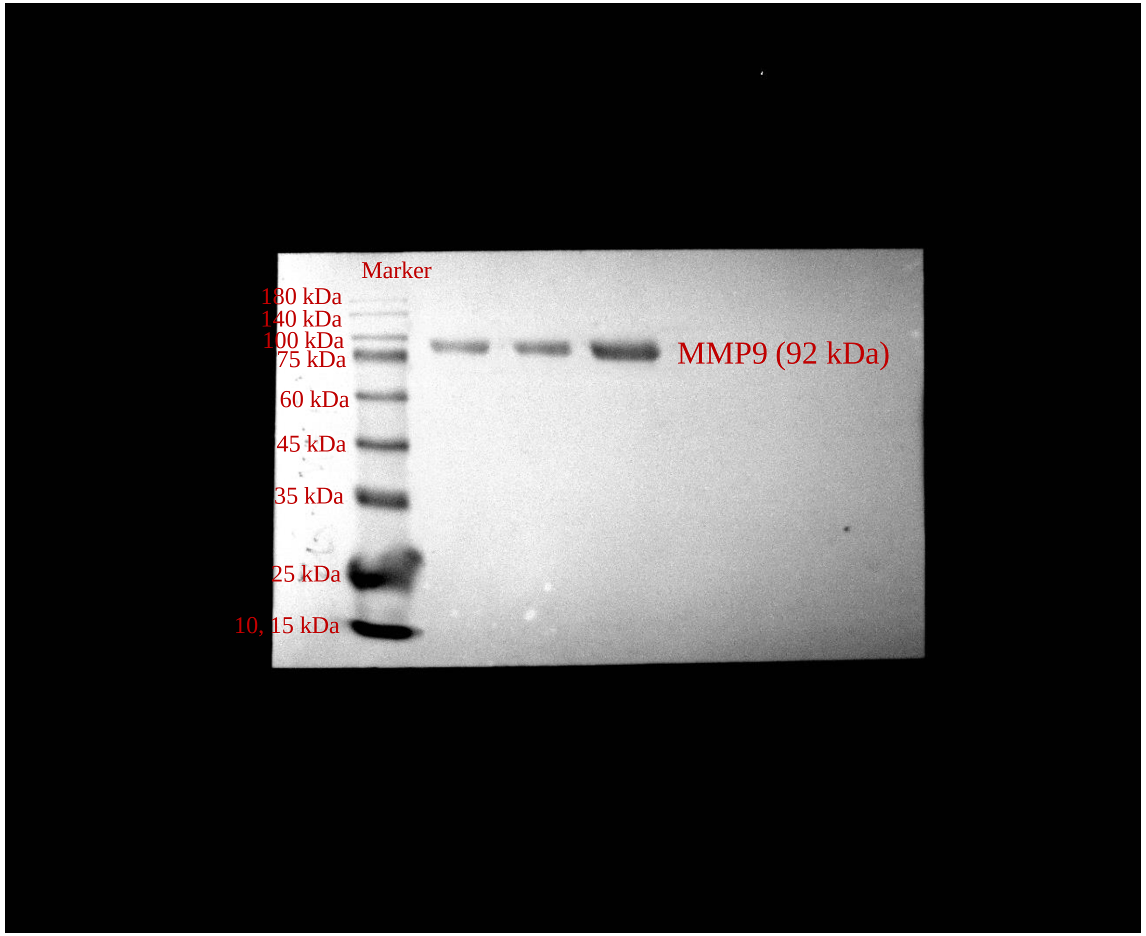

Marker
180 kDa
140 kDa
100 kDa
75 kDa
60 kDa
45 kDa
35 kDa
25 kDa
10, 15 kDa
MMP9 (92 kDa)

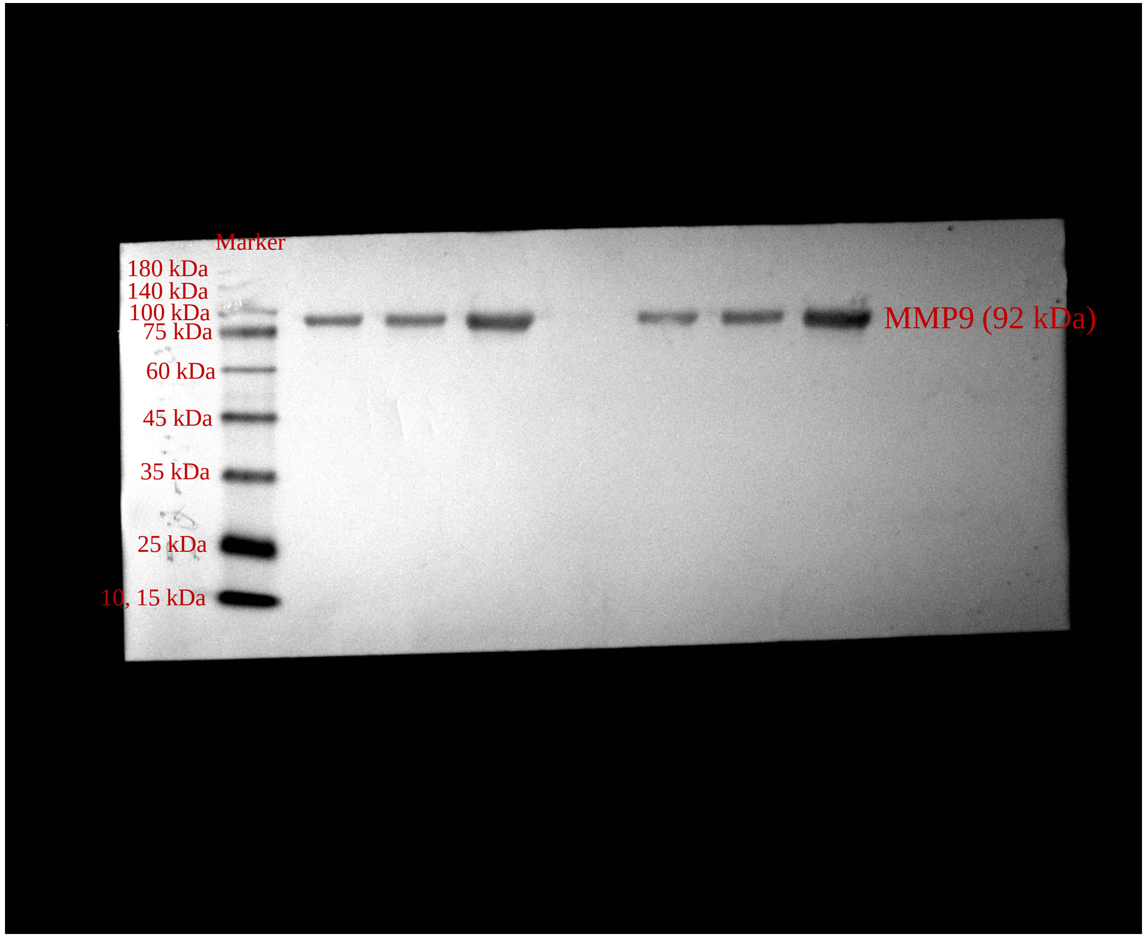

Marker
180 kDa
140 kDa
100 kDa
75 kDa
60 kDa
45 kDa
35 kDa
25 kDa
10, 15 kDa
MMP9 (92 kDa)

Fig.4B: β-actin

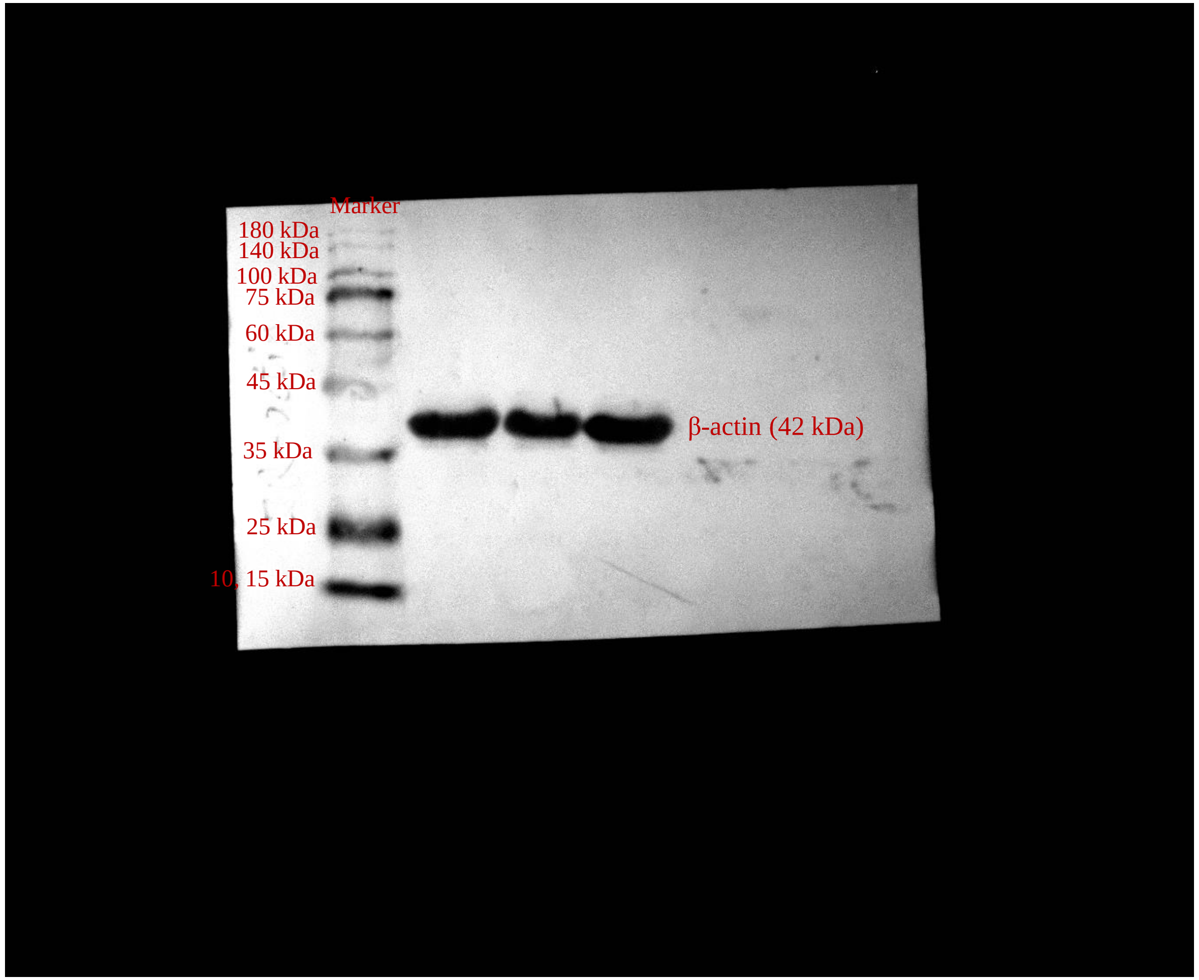

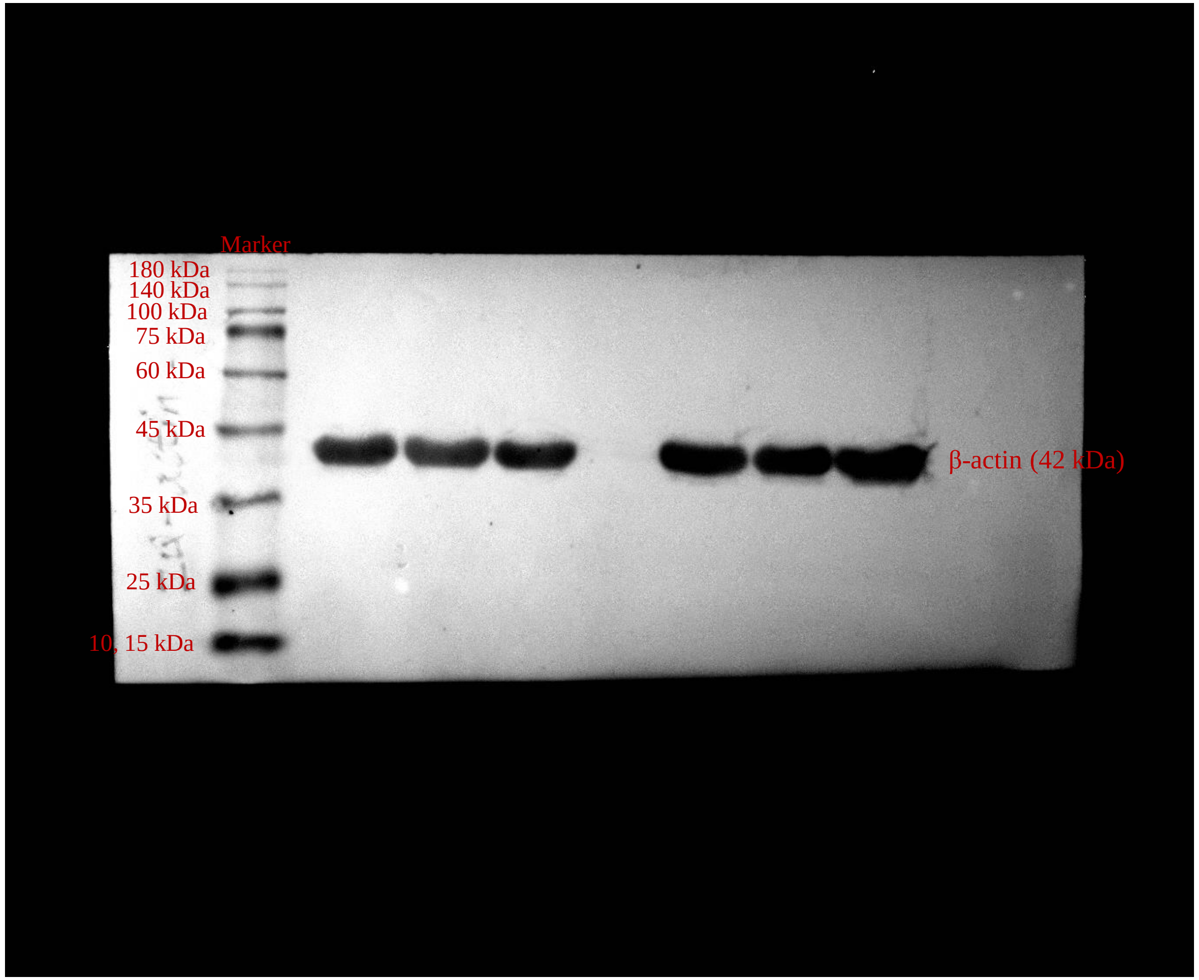

Fig.6E: MMP9

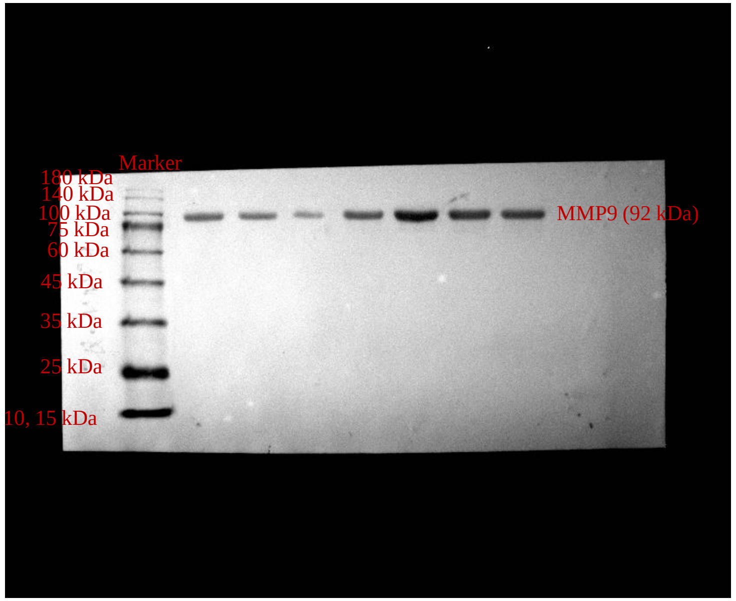
Marker
180 kDa
140 kDa
100 kDa
75 kDa
60 kDa
45 kDa
35 kDa
25 kDa
10, 15 kDa
MMP9 (92 kDa)

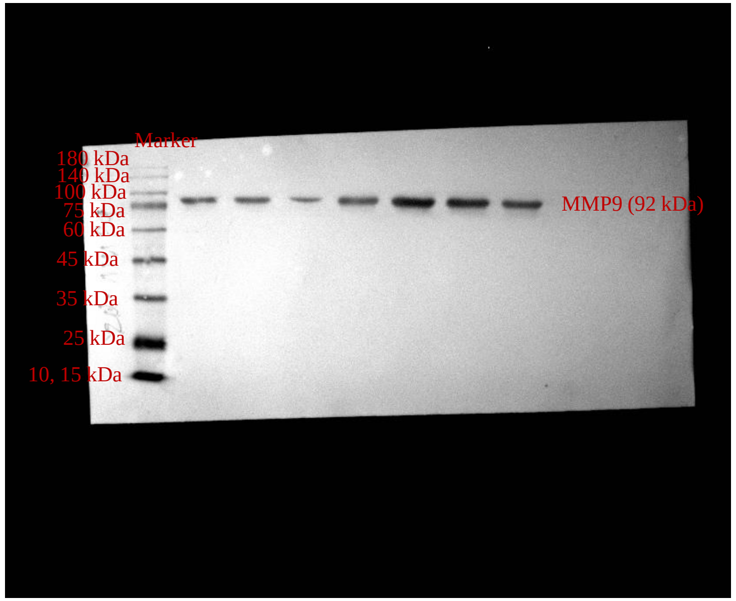
Marker
180 kDa
140 kDa
100 kDa
75 kDa
60 kDa
45 kDa
35 kDa
25 kDa
10, 15 kDa
MMP9 (92 kDa)

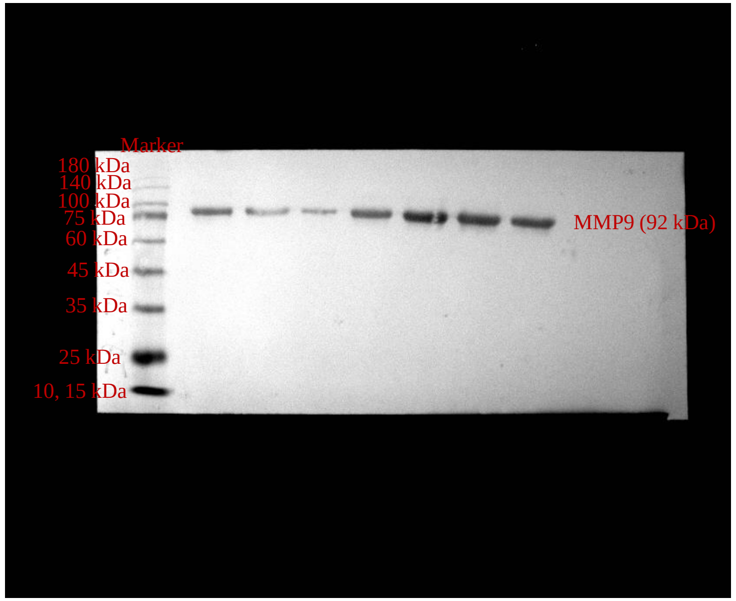
Marker
180 kDa
140 kDa
100 kDa
75 kDa
60 kDa
45 kDa
35 kDa
25 kDa
10, 15 kDa
MMP9 (92 kDa)

Fig.6E: β-actin

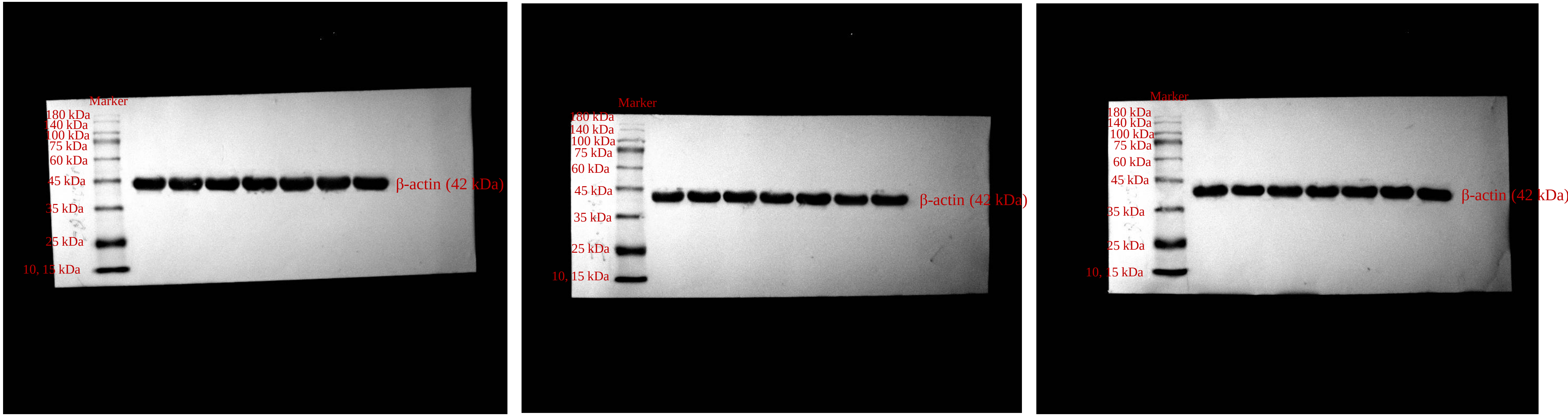

Fig.8G: MMP9

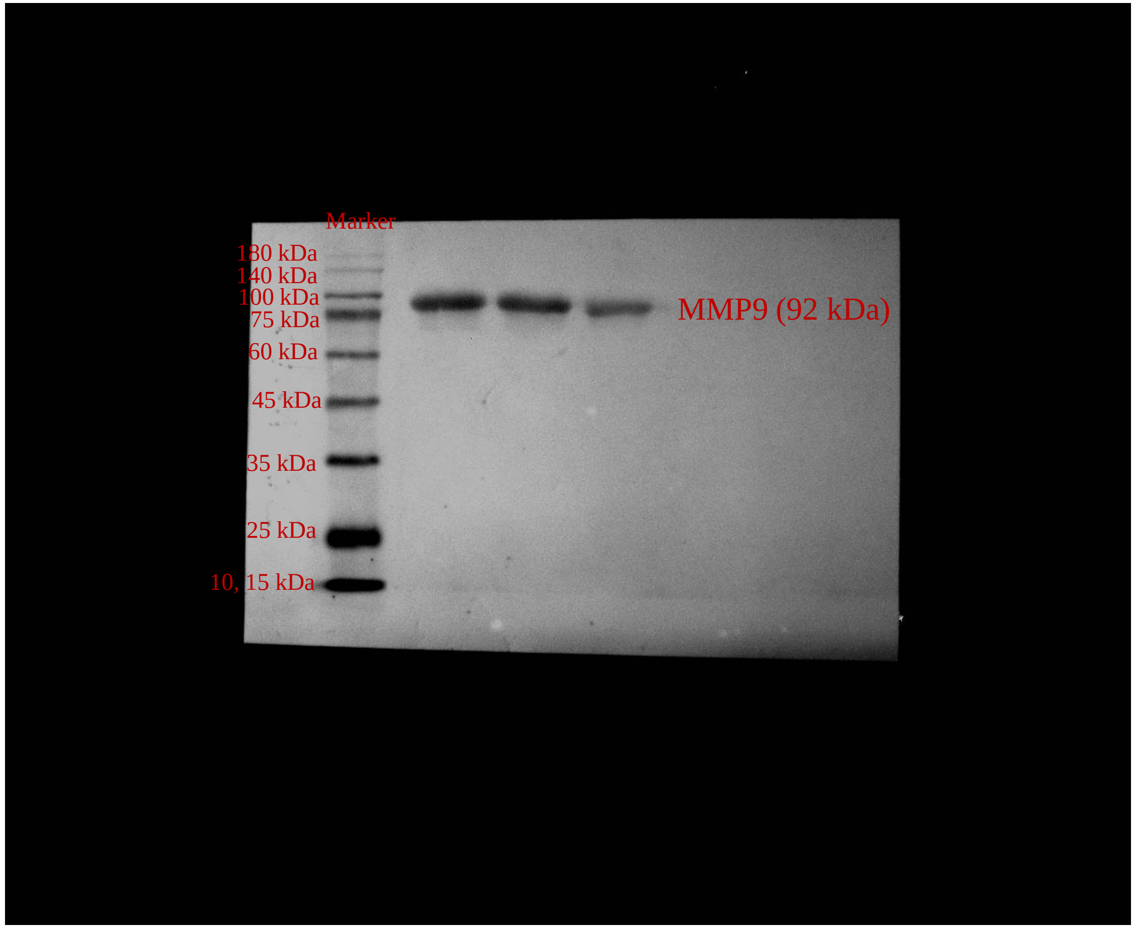
Marker
180 kDa
140 kDa
100 kDa
75 kDa
60 kDa
45 kDa
35 kDa
25 kDa
10, 15 kDa
MMP9 (92 kDa)

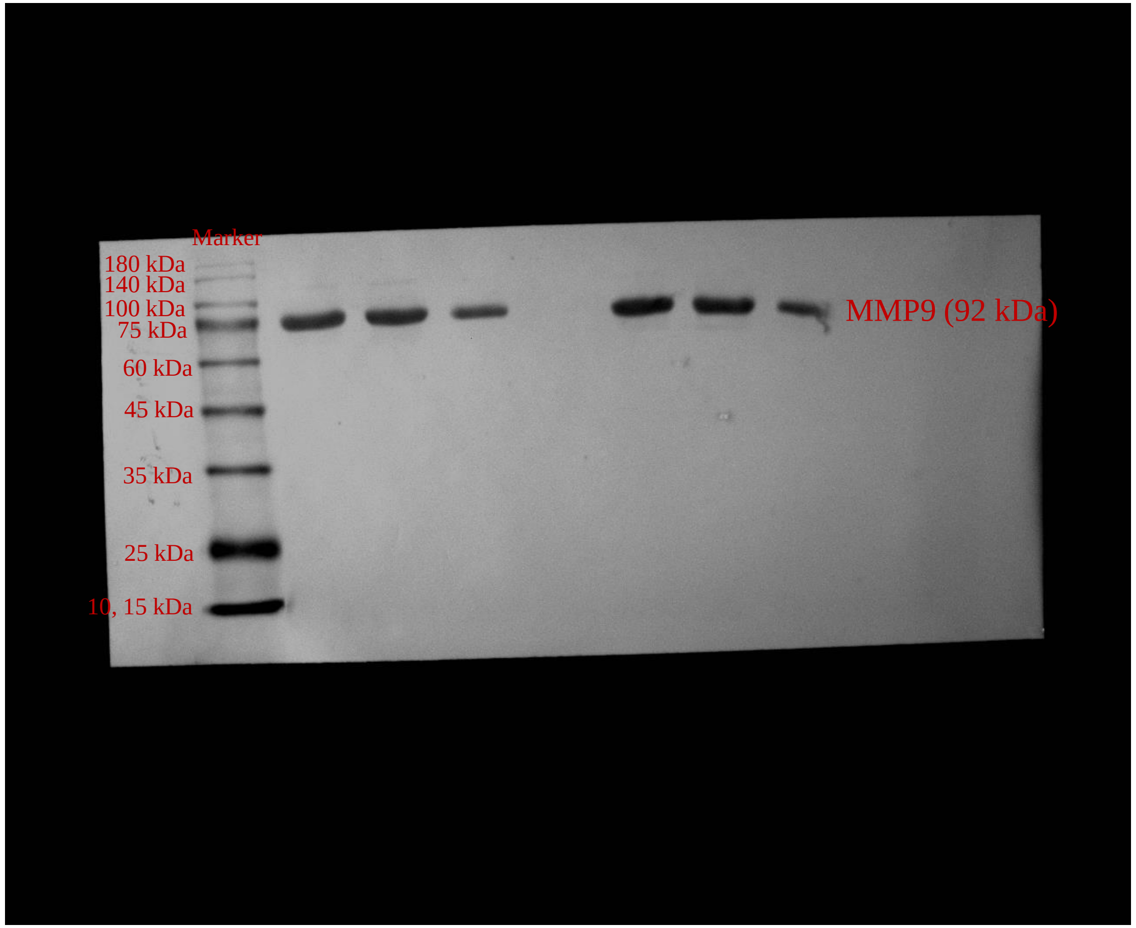
Marker
180 kDa
140 kDa
100 kDa
75 kDa
60 kDa
45 kDa
35 kDa
25 kDa
10, 15 kDa
MMP9 (92 kDa)

Fig.8G: β-actin

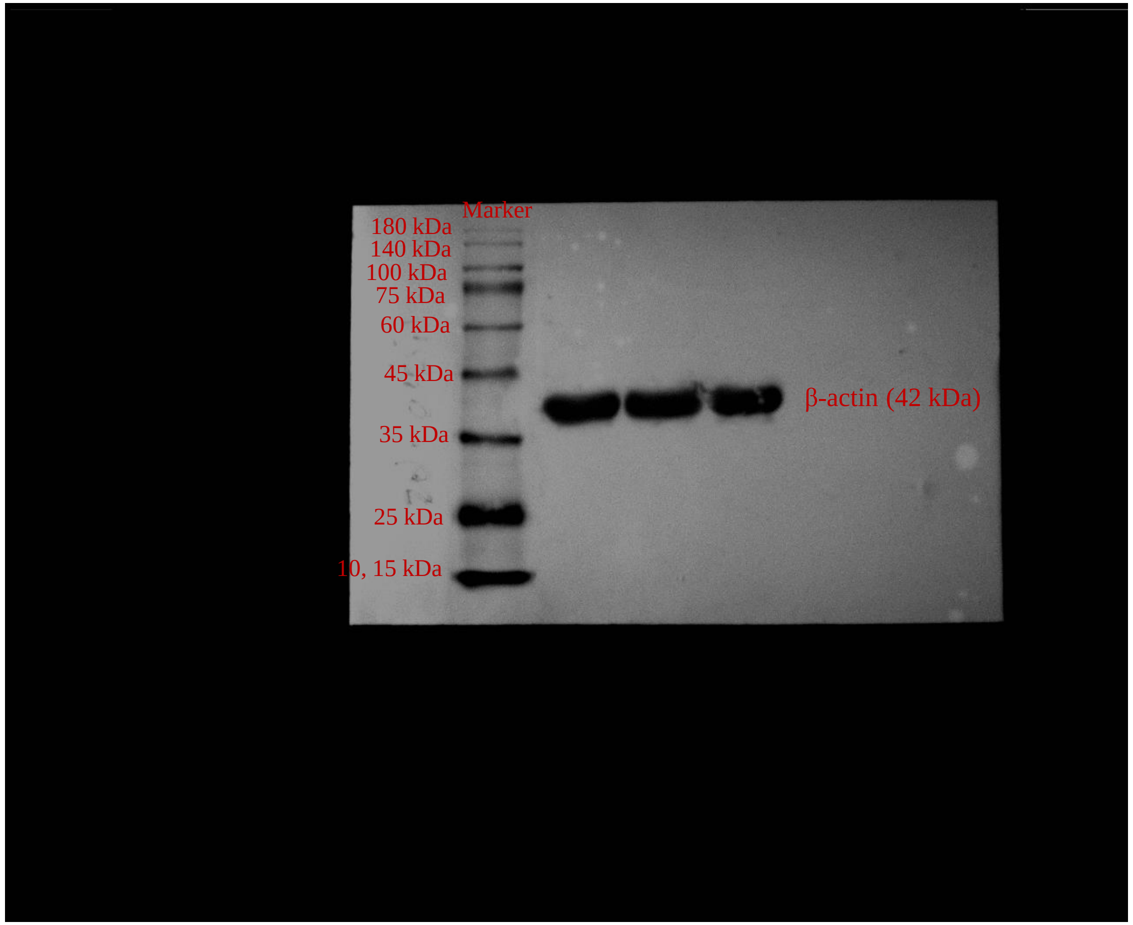

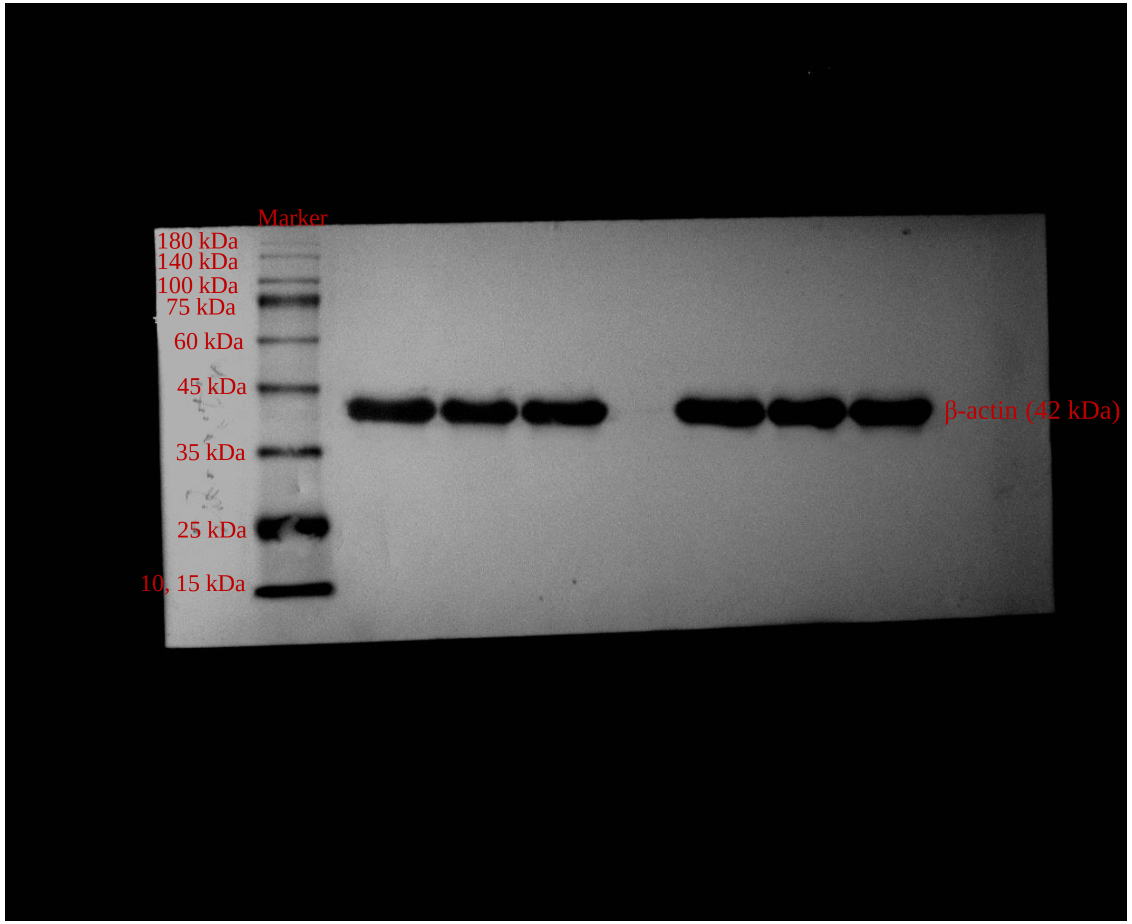

Supplement: Supplementary Materials — Supplementary File 1. Whole western blot bands for all figures. [file 7630698.f1.pdf]
